# Advances in Natural Product Extraction Techniques, Electrospun Fiber Fabrication, and the Integration of Experimental Design: A Comprehensive Review

**DOI:** 10.3390/molecules28135163

**Published:** 2023-07-02

**Authors:** Juthaporn Ponphaiboon, Wantanwa Krongrawa, Wah Wah Aung, Nawinda Chinatangkul, Sontaya Limmatvapirat, Chutima Limmatvapirat

**Affiliations:** 1 Department of Industrial Pharmacy, Faculty of Pharmacy, Silpakorn University, Nakhon Pathom 73000, Thailand; augusto_sc@hotmail.co.th (J.P.); krongrawa_w@su.ac.th (W.K.); wahwahaung31@gmail.com (W.W.A.); limmatvapirat_s@su.ac.th (S.L.); 2 Pharmaceutical Biopolymer Group (PBiG), Faculty of Pharmacy, Silpakorn University, Nakhon Pathom 73000, Thailand; nawinda.chi@siam.edu; 3 Faculty of Pharmacy, Siam University, Bangkok 10160, Thailand

**Keywords:** natural product, extraction, hyphenated technique, experimental design, electrospinning

## Abstract

The present review explores the growing interest in the techniques employed for extracting natural products. It emphasizes the limitations of conventional extraction methods and introduces superior non-conventional alternatives, particularly ultrasound-assisted extraction. Characterization and quantification of bioactive constituents through chromatography coupled with spectroscopy are recommended, while the importance of method development and validation for biomarker quantification is underscored. At present, electrospun fibers provide a versatile platform for incorporating bioactive extracts and have extensive potential in diverse fields due to their unique structural and functional characteristics. Thus, the review also highlights the fabrication of electrospun fibers containing bioactive extracts. The preparation of biologically active extracts under optimal conditions, including the selection of safe solvents and cost-effective equipment, holds promising potential in the pharmaceutical, food, and cosmetic industries. Integration of experimental design into extraction procedures and formulation development is essential for the efficient production of health products. The review explores potential applications of encapsulating natural product extracts in electrospun fibers, such as wound healing, antibacterial activity, and antioxidant properties, while acknowledging the need for further exploration and optimization in this field. The findings discussed in this review are anticipated to serve as a valuable resource for the processing industry, enabling the utilization of affordable and environmentally friendly, natural, and raw materials.

## 1. Introduction

Researchers are increasingly interested in the use of plant-derived, bioactive extracts due to the beneficial effects of their phytochemical constituents on human health. Phytochemical constituents, primarily polyphenols, are synthesized as secondary metabolites by plants through diverse metabolic pathways within plant cells [1]. Additionally, the extraction of bioactive compounds from animal tissues, such as beef fat and deer antler, remains popular, necessitating specialized extraction procedures. Extraction is a technique utilized in natural product research to separate bioactive compounds from various natural materials. Both plant and animal tissues are capable of yielding a variety of bioactive extracts, which can be prepared using a vast array of extraction techniques.

Conventional extraction techniques, including Soxhlet extraction, maceration, percolation, and decoction, are known to be time-, solvent-, and energy-intensive [2]. In contrast, various non-conventional techniques for the extraction process, such as supercritical fluid extraction (SFE), microwave-assisted extraction (MAE), and ultrasound-assisted extraction (UAE), have been developed to increase the yield of bioactive compounds and address the aforementioned issues [2]. These contemporary techniques have demonstrated their sustainability compared to conventional ones. Recently, low-temperature and environmentally friendly extraction techniques have gained popularity for producing high-quality bioactive extracts. UAE, in particular, has attracted considerable interest due to its advantages over conventional extraction methods, which include greater extraction efficiency, preservation of bioactive compound stability, shorter extraction time, and industrial application [3]. Thus, non-conventional extraction techniques like SFE, MAE, and UAE offer sustainable and efficient alternatives to conventional methods, allowing for increased bioactive compound yield, shorter extraction times, and improved stability, making them popular choices for high-quality extraction in low-temperature and environmentally friendly approaches.

The application of experimental design in the extraction of natural products offers significant advantages. It allows for the systematic study and optimization of parameters that affect the extraction process, resulting in various benefits such as optimized parameter settings, cost and time savings, improved data quality, enhanced extraction efficiency, deeper process understanding, and robustness testing. By utilizing experimental design, statistical analysis, and validation studies, researchers can identify the optimal conditions for maximizing extraction efficiency [4]. This technique is applicable to numerous industries, including the pharmaceutical, nutraceutical, and cosmetic industries. The following examples illustrate the use of experimental design in these fields, such as the use of factorial design to improve the solubility of compounds, orthogonal design to test the robustness of an analytical method for phytochemical analysis, and multivariate design to select the solvent in the development of analytical methods [5]. Therefore, the application of experimental design provides significant advantages, making it a valuable technique in the development of natural products.

In the case of utilizing natural products, the bioactive constituents of the extracts are characterized and quantified via chromatography coupled with spectroscopy to confirm their bioactive efficacy. High-performance liquid chromatography (HPLC) and gas chromatography (GC) are common techniques used for separating substances. For identification purposes, ultraviolet/visible spectroscopy (UV/Vis), infrared spectroscopy (IR), nuclear magnetic resonance spectroscopy (NMR), and mass spectroscopy (MS) are often employed [6]. Method development and validation are crucial for accurately quantifying biomarkers in natural product extracts due to the accuracy and precision offered by these techniques [7]. Consequently, chromatography combined with spectroscopy techniques plays a crucial role in the characterization and quantification of bioactive constituents in natural product extracts, allowing for accurate identification and reliable quantification of biomarkers for method development and validation purposes.

At the beginning of this article, we discuss both conventional and non-conventional extraction methods, particularly UAE; the parameters affecting the quality of UAE extracts; the techniques for separation and chemical characterization of chemical constituents in extracts; the quality control, standardization, and biological activity of extracts; and provide examples of the extraction and chemical analysis of natural products.

Incorporating optimized extracts with high concentrations of bioactive components into final products yields greater health benefits compared to crude extracts. In parallel, electrospinning, a versatile and innovative technique, is employed for fabricating nanofibers. It involves applying an electric field to a polymer solution or melt, which stretches and elongates the polymer into ultrafine fibers. These fibers exhibit nanometer to micrometer diameters, resembling the structure of natural extracellular matrix components and providing a high surface area-to-volume ratio. Electrospun fibers containing natural products show great promise in the pharmaceutical industry, offering unique opportunities for controlled and targeted drug delivery as well as improved bioavailability [8]. Thus, optimizing extracts with high concentrations of bioactive components and incorporating them into electrospun fibers can increase the product’s value.

The efficiency of electrospun fabrication is influenced by various variables. Extensive utilization of minimal experimental trials in combination with experimental design enables the evaluation of independent parameters’ effects on responses. Screening and optimization studies yield design models that approximate reality and help determine optimal settings for subsequent validation [8]. Continued research and development in this field are anticipated to unlock the full potential of electrospinning technology for the pharmaceutical industry, benefiting patients and healthcare professionals.

Electrospinnable polymers encompass a class of polymers possessing the requisite physical properties for processing through the electrospinning technique. Both synthetic polymers and natural polymers are commonly employed as electrospinnable materials. Selecting appropriate electrospun polymers [9] requires evaluating their physical and chemical properties, considering biocompatibility for biomedical applications, identifying desired properties, ensuring stability under processing conditions, considering availability and cost, and reviewing pertinent literature and studies. By taking these factors into account, the selection of electrospun polymers corresponds to the intended properties and application requirements. This article highlights shellac, a natural polymer, used in the development of electrospun fibers. It also explores the parameters that affect the quality of electrospun fibers, emphasizing their unique properties for various applications.

Overall, this review provides an overview of several extraction techniques for natural products, including their principles, advantages, limitations, and applications. It also explores the identification and quantification of chemical constituents and the development of electrospun formulations incorporating bioactive extracts to achieve specific objectives. The article highlights the use of UAE and electrospinning techniques for extracting bioactive components from natural sources and encapsulating them within electrospun polymeric fibers. This results in enhanced functionality, controlled release, and protection against the degradation of bioactives. Moreover, the article discusses the application of experimental design in the UAE procedure and electrospun fiber production. It emphasizes the utilization of various experimental designs to optimize the extraction process and enhance the quality of formulations. The successful encapsulation of natural product extracts in electrospun fibers is demonstrated, showcasing their potential applications in wound healing, antibacterial activity, and antioxidant properties. 

In general, review articles often provide detailed explanations and examples of specific knowledge, which may result in a limited understanding of the comprehensive process of product development using natural extracts. This limitation can hinder progress in research in this field. However, this article aims to address this issue by consolidating knowledge gained from our direct research experience in developing electrospun fibers from natural substances. The article covers a wide range of topics, starting from the initial stages of extracting compounds from plants and animals, investigating the phytochemical constituents using hyphenated techniques, ensuring the quality control of natural extracts, and culminating in the preparation of fibers containing biologically active extracts. Special emphasis was placed on employing experimental design to optimize the extraction and fiber fabrication processes, leading to the identification of optimal conditions for these procedures. This resulted in the preparation of extracts with high concentrations of active substances and excellent bioactivity, as well as the fabrication of nanofibers containing bioactive extracts with favorable chemical and physical properties and enhanced efficacy. All the authors hope that this review article will contribute to advancements in natural product extraction techniques, electrospun fiber fabrication, and the integration of experimental design.

## 2. Extraction

Extraction is a procedure that uses selective solvents to separate the pharmaceutically active components of plant or animal tissues from the inactive or inert portions. Polar solvents (e.g., water, ethanol, and methanol), intermediate-polar solvents (e.g., acetone, methylene chloride, and ethyl acetate), and nonpolar solvents (e.g., n-hexane, ether, and chloroform) are frequently used in the extraction of natural products [10]. The majority of the active ingredients in a crude extract tend to act synergistically. Purification of biologically active substances is not always required. To save time and expense on purifying bioactive compounds from crude extracts, the standardized extract can be used as the formula’s active ingredient [11]. The quality and quantity of bioactive constituents found in plant and animal materials are largely dependent on the selection of an appropriate extraction technique. During the extraction process, the solvents move into the solid raw materials and solubilize compounds with similar polarity in accordance with the principle of like dissolves like. In addition to employing an appropriate extraction method, the selection of an appropriate solvent is crucial. Moreover, extraction methods are continually modified [12].

There are two broad categories of extraction techniques: conventional and non-conventional (modern technique). Soxhlet, maceration, percolation, decoction, and hydro-distillation are the most frequently employed conventional extraction techniques in small research areas or Small and Medium-Sized Enterprises (SMEs) manufacturing facilities. However, these techniques have a variety of disadvantages, including lengthy extraction times, high costs, low extraction selectivity, poor extraction efficiency, high solvent consumption, and bioactive compound degradation due to prolonged exposure to high extraction temperatures. To overcome the limitations of conventional extraction methods, novel extraction techniques such as SFE, MAE, and UAE have been developed. These techniques can increase separation efficiency, reduce the use of raw materials, solvents, and energy, and have minimal environmental impact. In addition, the use of “green” solvents, which have numerous advantageous properties, such as being completely biodegradable, recyclable, noncorrosive, noncarcinogenic, and nonozone-depleting, enables the production of extracts that are recognized as safe and preferred by consumers [13].

In the present day, a variety of extraction techniques and tools are accessible. Consequently, the purpose of this review is to describe and compare the commonly used extraction methods based on their principles, strengths, and limitations in order to evaluate their suitability and practicability.

### 2.1. Conventional Extraction Methods

#### 2.1.1. Soxhlet Extraction

Soxhlet extraction is the most effective technique for the continuous extraction of solid raw materials using a hot solvent. Except for the extraction of thermolabile compounds, Soxhlet extraction is a general and well-established technique with superior performance to other conventional extraction techniques. The powdered solid substances are placed in a thimble within the Soxhlet apparatus. A round-bottomed flask containing the solvent and a reflux condenser is attached to the apparatus. The Soxhlet extraction procedure is as follows: (1) The solvent is heated by reflux. (2) Solvent vapor ascends the side tube, condenses in the condenser, and falls into the thimble. (3) Extraction of the solid matrix with a fresh solvent (4) From the extraction chamber, solutes are transferred to the reservoir. (5) The procedure is repeated until the extraction is complete [14].

#### 2.1.2. Maceration

Maceration is a method of solid–liquid extraction. Traditionally, maceration was used to recover bioactive compounds from plant or animal materials by combining solvents with or without heat and agitation or shaking to enhance mass transfer and the solubility of compounds. The solvent is added to the closed vessel containing the powdered solid matrix. A long time is allowed for extraction (varying from several hours to days), and a sufficient amount of time is allowed for the solvent to diffuse through the plant cell wall or animal tissue in order to solubilize the chemical constituents present in those materials. The process occurs solely via molecular diffusion [15].

#### 2.1.3. Percolation

The most common technique for extracting active ingredients for the preparation of tinctures and fluid extracts is percolation. Typically, a percolator (a narrow, cone-shaped vessel with open ends) is utilized. The powdered solid materials are moistened with an adequate amount of solvent and allowed to stand for several hours in a tightly sealed container before being packed, and the percolator’s top is sealed. An additional solvent is added to form a thin layer above the mass, and the mixture is macerated for an extended period of time in a closed percolator. The percolator’s outlet is then opened, and the liquid extract is allowed to slowly drip. Additional solvent is added as necessary until the percolate reaches approximately three-quarters of the required volume. The extract is then pressed, the required volume of solvent is added to the percolate, the combined liquid is clarified by filtration or by standing, and then decanted [11].

#### 2.1.4. Decoction

A decoction is a water-based preparation that can be used to extract water-soluble compounds from medicinal plant material. When dealing with tough, fibrous plants, barks, and roots, decoction is the preferred method. In this procedure, the plant material is typically reduced to small fragments or powder for effective dissolution. The liquid extract is produced by boiling plant material in water. On occasion, aqueous ethanol or glycerol can be substituted for water. The amount of water used in this method is dependent on the plant material’s hardness [12].

### 2.2. Non-Conventional Extraction Methods

#### 2.2.1. Microwave-Assisted Extraction (MAE)

Microwaves are electromagnetic waves with a frequency between 300 MHz and 300 GHz that are non-ionizing [16]. Ionic conduction and dipole rotation, which are most frequently present simultaneously, are two phenomena that govern microwave heating because of their direct impact on polar materials and solvents. Ionic conduction is the electrophoretic migration of ions under the influence of an alternating electric field. The solution’s resistance to the migration of ions generates friction, which eventually causes the solution to heat up. Dipole rotation is the realignment of a molecule’s dipoles in a rapidly varying electric field. Microwaves produce heat by interacting with polar compounds in the plant and animal matrix, such as water and certain organic molecules, via ionic conduction and dipole rotation [12,17]. Both ethanol and methanol will absorb less microwave energy than water, but their overall heating efficiency will be greater than that of water. On the other hand, hexane, and other less polar solvents (low dielectric constant), such as chloroform, are microwave-transparent and do not generate heat [16]. In MAE, heat and mass transfer occur in the same direction, producing a synergistic effect that accelerates extraction and increases extraction yield. Evaporation of water increases the pressure within plant and animal cells, resulting in swelling and eventual rupture. This facilitates the leaching of constituents from ruptured cells into the surrounding solvent. MAE can also be carried out without a solvent. In a dry or solvent-free MAE, the sample water acts as a solvent and enables cell lysis. Aromatic plants have been extracted of their essential oils and antioxidants using solvent-free MAE. Numerous variables, such as microwave power, duration, dielectric properties of the sample mixture, and type of solvent, can be modified to achieve the desired extract characteristics [12,17].

#### 2.2.2. Supercritical Fluid Extraction (SFE)

A fluid in conditions of pressure and temperature beyond its critical point is known as a “supercritical fluid,” which is a dense fluid with characteristics between those of a gas and a liquid. Its viscosity and diffusivity are similar to those of a gas, while its density is comparable to that of a liquid. Consequently, a supercritical fluid can function as a liquid-like solvent, except with enhanced mass transfer kinetics [18]. SFE relies on the solubilizing properties of supercritical fluid, which can be obtained by applying pressure and temperature above the critical point of the sample mixture. Although carbon dioxide (CO_2_) is the favored extraction solvent for nonpolar substances, the polarity of supercritical carbon dioxide (SC-CO_2_) can be increased by adding a miscible polar solvent (like hexane, methanol, ethanol, isopropanol, and dichloromethane) as a modifier or co-solvent. Ethanol is recommended as a co-solvent due to its lower toxicity and miscibility with CO_2_. Due to the properties of SC-CO_2_, which include high selectivity, relative affordability, safety, non-flammability, non-corrosiveness, inertness (least alteration of bioactive substances to maintain their therapeutic or functional properties), and the capability to extract thermally unstable compounds, it is extensively used to extract many natural products, including non-polar compounds such as lipids and volatile oils, by optimizing the extraction pressure and temperature [12].

#### 2.2.3. Ultrasound-Assisted Extraction (UAE)

Ultrasound refers to any sound wave or acoustic energy that exceeds the human hearing threshold of 20 kHz. For the extraction of natural products, ultrasonication treatments typically range from 20 to 100 kHz and 10 to 1000 W/cm^2^ in power density. The ultrasonic wave travels through large quantities of materials based on their mechanical and physical characteristics, such as their composition and structure. As a result of the ultrasonic waves’ combination of compression and rarefaction, molecules are displaced from their original position [19]. The UAE principle relies on cavitation effects, which enhance mass transfer, and close interaction between the solvent and plant or animal tissues. The cavitation bubbles are formed when the acoustic pressure produced by rarefaction cycles exceeds the attractive forces of the liquid medium’s molecules. The cavitation bubbles will expand and contract during the rarefaction and compression cycles if the acoustic pressure is totally inadequate. If the bubbles do not attain the critical size for implosion or collapse, the phenomenon is referred to as stable cavitation, which lessens the cavitation effect. Alternatively, if the pressure is high enough, transient cavitation generates a great deal of energy, and the micro-bubbles continue growing until collapsing or imploding at the critical size. The collapse of cavitation bubbles near sample tissue surfaces generates microjets, causing tissue disruption and deep solvent penetration into the matrix of the tissue. The release of thermal energy facilitates mass transfer. During UAE, cavitation, thermal, and mechanical phenomena related to ultrasonic energy cause cell wall disruption, particle size reduction, plant matrix destruction, and enhanced mass transfer without affecting the chemical structures and biological activity of chemical constituents in the extracts. Ultrasound eliminates the need for high temperatures during the extraction process, allowing the UAE to protect heat-sensitive compounds [13].

The ultrasonic devices used in the UAE can be briefly categorized into three groups based on their mode of action: ultrasonic bath mode, sonotrode (ultrasonic probe) mode, and a special-design mode derived from the first two modes [20]. The ultrasonic cleaning bath is the most commonly used and readily available method. Typical components include a transducer, a container, a timer, and a heater. The sound energy derived from the electric current and converted by the transducers can be transferred into the bath’s medium. The extraction vessel in small and lab-scale UAE is typically a flask or tube that is partially submerged in water and held in a specific position to maximize cavitation. The ultrasonic bath is considered the extraction vessel at larger scales. A mechanical stirrer is always required in this circumstance to prevent the matrix from floating or sinking and escaping the ultrasonic irradiation. From the foregoing, it is clear that bath sonication is an indirect contact method employing an ultrasonic bath of high intensity. A water bath receives ultrasonic energy, which is then transferred to a vessel or sample container. A probe that contacts the samples is unnecessary for a bath sonicator. This method can therefore prevent cross-contamination [21,22]. Regarding the ultrasonic probe mode, a typical system consists of an ultrasonic probe, a container that can be connected to the probe, and occasionally a stirrer and temperature control system. For larger-scale extraction with a probe-type device, the special-design mode employs a more powerful ultrasonic probe as well as sufficiently large bores and valves in the appropriate positions to allow the matrix and solvent to pass [20]. In order to obtain greater ultrasonic energy, a probe is inserted into a mixture of sample and solvent and then comes into direct contact with the sample materials. Thus, cross-contamination between samples is possible due to direct probe contact [21,22]. The difference between ultrasonic bath mode and ultrasonic probe mode is shown in Table 1.

The advantages and disadvantages of common conventional and unconventional extraction techniques are summarized in Table 2 based on the preceding discussion.

### 2.3. Parameters Affecting the Quality of Extracts Obtained from UAE

Numerous parameters, such as yields, phytochemical constituents, and biological properties, influence the extraction conditions necessary to obtain the desired quality and quantity of extract during the extraction of plant materials. Important parameters for obtaining high-quality active ingredients for pharmaceutical preparations include the selection of appropriate extraction techniques. This article asserts that ultrasound waves can disrupt biological cell walls and facilitate the release of bioactive components during extraction. Major factors contributing to the improvement of extraction with ultrasound-assisted techniques as compared to those that do not utilize ultrasound waves are efficient cell disruption and mass transfer. UAE is considered an environmentally friendly extraction method due to its low cost, simplicity, efficiency, high extraction yield, minimal energy and solvent requirements, and low operating temperature [19,25,26]. Due to the heat generated by ultrasound waves and the free radicals produced by high-energy ultrasound waves, it is challenging to control the temperature with UAE [27]. Numerous variables affect the UAE, including solvent type and concentration, extraction temperature and time, solvent-to-solid ratio, solid sample particle size, and ultrasound frequency. Because of the various factors influencing the UAE, experimental design should be employed to determine the optimal conditions for obtaining the high concentration of the desired compound [28].

Despite the fact that the UAE technique reveals distinct procedures and advantages in comparison to conventional extraction methods, several parameters affecting the quality of extracts must be considered in order to maintain and improve extraction efficiency.

#### 2.3.1. Frequency

It is important to note that ultrasonic frequency is a critical parameter that can regulate the physical effects of burst bubbles [29]. Due to the inverse relationship between ultrasonic frequency and rarefaction phase duration, the implosion of cavitation bubbles is hampered at high frequencies. More mass transfer resistance is provided by the huge number of bubbles generated at high frequency [3]. For the most part, the shear and mechanical force produced by ultrasound with a low frequency and high intensity (ultrasonic power) is desirable for UAE [30].

#### 2.3.2. Power and Amplitude

The rate at which sound energy is emitted per unit of time is called ultrasound power, and it varies depending on the mass of the solvent, the solvent’s heat capacity, and the time interval over which the power is measured [25]. The transfer of energy to the extraction liquid during UAE has also been quantified in terms of amplitude percentage. Increases in power and amplitude cause greater vibration in the solvent medium, which in turn increases the solvent’s penetration and extraction efficiency [31].

#### 2.3.3. Solvent

The polarity of the solvent is one of the factors influencing the type and quantity of extracted molecules as well as the extraction yield. Various solvents with high and low polarity, such as water, alcohols (ethanol and methanol), acetone, etc., have been used in the UAE method to extract numerous bioactive compounds. The selection of the solvent depends primarily on the solubility of the required substances [32].

#### 2.3.4. Processing Time

Increasing sonication time has an impact similar to increasing power and temperature. Increasing sonication duration initially increases yield, but when time is extended further, yield decreases. In response to an initial increase in sonication time, the cavitation impact of the ultrasound increased the tissue matrix’s swelling, breakdown, and pore creation. All of these attributes increase the exposure of the solute and solvent, resulting in a better extraction yield. However, prolonged ultrasound exposure causes structural damage to some solutes and diminishes the extraction yield, especially due to oxidative degradation [33]. Consequently, the processing time should be optimized in order to maximize extraction efficiency and reduce the oxidative effects caused by the ultrasound wave.

#### 2.3.5. Extraction Temperature

Similar to the effect caused by an increase in power and amplitude, an initial increase in temperature increases the extraction yield, but further increases in temperature decrease the yield. Increasing the temperature decreases the solvent’s viscosity, which increases the solvent’s diffusivity in the tissue matrix, resulting in a greater extraction yield [3]. The formation of cavitation bubbles is influenced by temperature and solvent type, resulting in cell rupture and subsequent release of bioactive compounds [34]. The yield decreases as the temperature rises due to the weakened cavitation effect. Moreover, an extraction temperature that is too high may degrade thermolabile compounds [35].

#### 2.3.6. Solvent-to-Solid Ratio

The solvent-to-solid ratio is the volume of solvent to the amount of sample extracted [3]. The extraction yield increases with increasing solvent-to-solid ratio until a particular limit is reached, after which it decreases. At low solvent-to-solid ratios, the viscosity of the sample-solvent mixture is high, which makes the cavitation effect more difficult because the negative pressure in the rarefaction cycle must achieve a greater cohesive force in the high-viscosity mixture. With an increase in the initial ratio of solvent to solid, the viscosity and concentration of the mixture decrease, resulting in a greater cavitation effect. A high ratio of solvent to solids increases yield by increasing fragmentation, corrosion, and pore formation. Increasing the material’s contact area with the solvent may also increase yield. The decrease in yield at extremely high solvent-to-solid ratios can also be attributed to the enhanced cavitation effect, which degrades the target compound [36].

## 3. Techniques for Separation and Chemical Characterization of Extracts

As part of the overall quality and biosafety assessment, chemical characterization of extracts involves the identification and quantification of the extract’s chemical components. The study of biological extracts derived from diverse plants, animals, or microorganisms differs from the study of isolated (pure) compounds. The extracts are typically multicomponent mixtures of active, partially active, and inert substances, and their activities are rarely exerted on a single target. It is commonly believed that multiple components in the extract can contribute to a synergistic response and that the therapeutic response is sometimes related to conventional preparation and administration. The composition of the extract varies based on the extraction technique and the raw materials employed (such as growing conditions, development stage, and harvesting protocols) [37]. Therefore, it is necessary to investigate both the type and quantity of bioactive substances in the extracts that have been prepared. In addition to the extraction parameters, a comprehensive evaluation of the phytochemical composition is required. Extracts should ideally be analyzed qualitatively and quantitatively using a variety of chromatographic and spectroscopic techniques. 

Thin-layer chromatography (TLC)-densitometry, for instance, is one of the most straightforward chromatographic separation techniques used for qualitative and quantitative analysis. Nevertheless, TLC can only detect substances that fluoresce under long-wavelength ultraviolet light (360 nm) or absorb under short-wavelength ultraviolet light (254 nm). Additionally, certain compounds can be detected by spraying reagents that undergo a chemical reaction with a compound on the TLC plate, transforming it into a colored compound [38]. In order to purify or isolate large quantities of natural products, preparative high-performance liquid chromatography (preparative HPLC) typically requires big columns, substantial sample loading, a high mobile phase volume, and elevated flow rates in an HPLC system. To obtain small amounts of pure compounds, it is sometimes possible to conduct isolation with analytical-scale HPLC systems. In general, bioassay screening requires small quantities of pure substances, whereas structure elucidation requires large quantities. Normal phase (suitable for separation of low-polar or lipophilic natural products), reversed phase (suitable for isolation of high-polar or hydrophilic natural products), size exclusion (suitable for separation of lower molecular weight compounds), and ion exchange (suitable for separation of amines or acids) are among the various HPLC modes currently available for purifying most classes of natural products [39]. Gas chromatography (GC) is one of the most essential and widespread techniques for the separation of natural products, especially terpenes and other high-temperature-stable volatile compounds. If natural products are nonvolatile, they must undergo silylation, acylation, alkylation, or other suitable derivatization reactions to make them volatile. Chromatographic techniques for GC are categorized as adsorption chromatography (interaction between a solid stationary phase and the gas phase) and partition chromatography (interaction between a liquid stationary phase and the gas phase). To accomplish a good separation in GC, the column must be carefully chosen based on the type of natural products, the number of crude extracts, and their complexity. For instance, polyethylene glycol used as the stationary phase of a GC column is appropriate for separating compounds with polar functional groups, such as organic acids and volatile oils [40]. As stated previously, crude extracts of natural products typically contain numerous compounds, and the purification of specific molecules requires a particular separation technique.

For structure elucidation or identification, ultraviolet-visible (UV-Vis) spectrophotometry, Fourier transform infrared (FTIR) spectroscopy, nuclear magnetic resonance (NMR) spectroscopy, and mass spectrometry (MS) has been utilized. The UV-Vis spectrophotometer provides information about the structure of conjugated or aromatic molecules. Different functional groups of natural products can be identified using FTIR spectra. One-dimensional (1D) NMR experiments, such as proton and carbon-13 NMR, convey information regarding the qualitative and quantitative composition of natural products. Initially, they are employed to ascertain the chemical structure of various classes of natural products, including amino acids, organic acids, sugars, phenolic compounds, alcohols, and esters [41]. In addition to 1D-NMR experiments, numerous 2D-NMR experiments have been developed for structure elucidation by providing information on the correlations of nuclei via scalar or dipolar couplings, revealing the connections between proton and proton and proton and carbon-13 or heteroatom within molecular structures [42]. Despite the fact that compounds are absolutely identified through the interpretation of 2D-NMR experiments, 1D-NMR experiments still provide valuable information. For example, the number of carbon-13 signals helps to identify the structures of terpenes made up of isoprenoid units; the increase in area under the proton signal and the decrease in carbon-13 signals help to identify symmetric molecules; and specific proton and carbon-13 chemical shifts help to confirm the presence of aromatic rings or carbonyl groups in molecular structures. In addition, proton NMR spectra disclose the presence of certain functional groups, including hydroxyl and primary and secondary amine groups [42,43]. Therefore, NMR can be used to guide the isolation of natural products. MS can determine the molecular weights and elemental compositions of compounds with diverse chemical and physical properties, making it essential for natural product structure elucidation. If a molecular ion is created with enough excitation energy, it will undergo a sequence of unimolecular processes to produce fragment ions with relative abundances specific to its structure. The mass spectra can reveal the functional groups and how they are linked to generate a unique molecular structure by analyzing fragmentation patterns [44]. Due to the inherent limitations of each detection method, however, the use of a single method may result in the detection of only a subset of components, leaving a large number of substances undetected. Consequently, it may be necessary in some instances to demonstrate the structures of natural products using multiple spectroscopy techniques.

Before chromatographic separation, sample preparation is crucial for the elimination of interferences and unintended substances. There are numerous techniques for sample preparation, including solid-phase extraction (SPE) and liquid-liquid extraction (LLE). In general, compounds of interest are extracted using LLE in order to separate compounds based on their relative solubilities in two distinct immiscible liquids, typically aqueous (polar) and an organic solvent (non-polar) [45]. LLE enhances the concentration of the substance of interest, resulting in a proportional increase in the ratio of the area beneath the proton NMR signal (integration value) in the structure of the substance of interest to that of other substances [46]. In addition to using proton NMR to elucidate the chemical structures of pure natural products obtained from chromatographic separations, it can also be used to monitor the substances of interest during sample preparation. For instance, the aqueous extract of the medicinal shrub *Tamarix gallica* was further extracted using ethyl acetate and n-butanol in LLE. A proton NMR phytochemical analysis revealed that the n-butanol extract was rich in phenolic compounds, particularly flavonoids, due to the presence of aromatic systems in the molecular structures [47].

Liquid or gas chromatography-coupled spectroscopic techniques such as high-performance liquid chromatography-ultraviolet (HPLC-UV), high-performance liquid chromatography-mass spectrometry (HPLC-MS), high-performance liquid chromatography-nuclear magnetic resonance (HPLC-NMR), gas chromatography-flame ionization detection (GC-FID), and gas chromatography-mass spectrometry (GC-MS) are hyphenated techniques that have been used to simultaneously separation and identification of the constituents of an extract [48,49,50]. These techniques permit the rapid determination of known natural products using a minute amount of sample material. To detect different classes of compounds using HPLC-UV, different detection wavelengths are required. With HPLC-UV analysis, only compounds containing chromophores can be detected [50]. HPLC-NMR is a viable approach for routinely analyzing complex extracts. The combination of carbon-13 NMR and proton NMR provides detailed structural information on all organic compounds. However, the method is costly, maintenance-intensive, and relatively insensitive [50,51]. Some compounds ionize only in the positive or negative ion mode of HPLC-MS and GC-MS; both ion modes are necessary to detect as much as possible. Certain substances may require unique ionization sources [52]. By employing different temperature gradients and stationary phases, GC-FID and GC-MS can exclude the co-elution of compounds. Nonetheless, it is only applicable to volatile or derivatized compounds [53]. 

It is well known that the chemical composition of natural extracts is complex. The structure elucidation and quantification of biologically active compounds in extracts necessitate specialized equipment, particularly the hyphenated technique. As mentioned above, combining a separation technique with an online spectroscopic detection technology produces the hyphenated technique [48,49,50,51,52]. The separation part, typically an HPLC or GC, and detection part, one or more spectroscopic detectors such as UV–vis, MS, and NMR, are coupled for simultaneous separation, structural identification, and quantification of components present in a complex extract [54]. As part of the optimization of extraction, the hyphenated technique was used to prove the chemical structure and analyze the content of active substances in the extract (experimental response). Examples of the implementation of hyphenated techniques to optimize extraction are provided in the subsections that follow.

### 3.1. High-Performance Liquid Chromatography-Diode Array Detection (HPLC-DAD)

The photodiode array (PDA) detector, also known as the diode array (DAD) detector, is an advanced type of UV-vis spectrophotometer that can be coupled to an HPLC to produce the HPLC-DAD. It is remarkably useful for analyzing natural compounds containing chromophores, such as polyphenols, flavonoids, and aromatic alkaloids. For each peak on an HPLC chromatogram, a PDA detector provides three-dimensional (3D) UV-vis data, typically consisting of UV-vis absorption spectra from 190 to 500 nm. The data are accessible in the domains of time, concentration, and wavelength. Therefore, it is possible to rapidly anticipate unique absorption regions corresponding to particular compounds, which represent the peak’s purity [54,55]. Organic solvents used as components of a mobile phase have UV absorption cutoff wavelengths. The solvent itself absorbs all light at wavelengths that are shorter than the cutoff. When selecting a solvent with significant UV absorption at the wavelength at which measurements are performed, complications arise. In such instances, the substance’s signal and the solvent’s signal will overlap, resulting in an inaccurate determination [54]. The following are examples of optimizing extraction using HPLC-DAD for the determination of bioactive compounds. The developed HPLC-DAD method provided a rapid and dependable method for identifying and quantifying bioactive scopoletin in a noni extract (*Morinda citrifolia* L). The optimal yield of scopoletin was obtained by extracting at an increased temperature of 60 °C for 12 min with ethanol as the extraction solvent and a solid-to-solvent ratio of 1:30 (*w*/*v*) [56]. To optimize extraction, a reversed-phase HPLC-DAD method with gradient elution was developed for quantifying five neuropharmacological flavonoids, including orientin, isoorientin, vitexin, isovitexin, and rutin, in a *Passiflora* species leaf extract. This method was extremely precise and accurate for all samples analyzed [57]. The simultaneous quantification of three methoxyflavone markers using a validated HPLC-DAD method in accordance with ICH guidelines was a suitable strategy for optimizing the extraction of *Kaempferia parviflora* rhizomes. [58]. From the foregoing, it is evident that HPLC-DAD is an ideal technique for the analysis of UV-absorbing compounds containing an aromatic ring and conjugation.

### 3.2. High-Performance Liquid Chromatography-Mass Spectrometry (HPLC-MS)

An HPLC-MS combines the chemical separation capability of an HPLC with the ability of a mass spectrometer to detect and confirm the molecular identity selectively. The sample eluted from an HPLC column is then accelerated through either a quadrupole or an ion trap mass analyzer, and the ions are identified according to their mass-to-charge (*m*/*z*) ratios. MS is one of the most sensitive and selective chemical analysis techniques, and it provides data on the molecular weight and fragmentation pattern of natural products. MS provides invaluable information for confirming the identities of natural products, particularly if they are known compounds [54,59]. In a mass spectrometer, matrix-assisted laser desorption/ionization (MALDI), electrospray ionization (ESI), and atmospheric pressure chemical ionization (APCI) are common ionization techniques. MALDI’s usefulness for analyzing heterogeneous samples makes it an attractive technique for the mass analysis of complex extracts. It can measure masses up to 300,000 Da. However, matrix background, which can be a problem for natural products with a mass of less than 700 Da. ESI and APCI are soft ionization techniques that can analyze a practical mass range of up to 70,000 Da and 2000 Da, respectively, with high sensitivity. However, ESI has a relatively low salt tolerance, whereas APCI may decompose samples prior to vaporization through thermal desorption [59,60]. Numerous studies have utilized HPLC-MS to study the optimization of extraction, as follows: To optimize grape seed phenol extraction, antioxidant-active phenolic acids, flavan-3-ols, and procyanidins were characterized using HPLC coupled to tandem mass spectrometry (HPLC–MS/MS). Because a single MS run provides rather limited information about the structures of the compounds, the advent of tandem mass spectrometry (MS/MS), which provides fragments through collision-induced dissociation of molecular ions, was used in this study. Furthermore, a significant contributor to selectivity is the stationary phase. The pentafluorophenyl (PFP) column exhibited greater resolution of chromatographic separation for isomeric phenols with identical m/z values as precursors and product ions than the C-18 and phenyl hexyl columns [61,62]. To demonstrate the efficacy of MAE, the phenolic compounds in olive leaf extracts obtained from experimental variables that influence the extraction process were analyzed using an HPLC coupled to electrospray time-of-flight mass spectrometry (ESI-TOF-MS) and electrospray ion trap tandem mass spectrometry (ESI-IT-MS/MS). The HPLC chromatogram of the analyzed extracts revealed multiple peaks that could be characterized by combining ESI-TOF-MS and ESI-IT-MS/MS. The results indicated the presence of apigenin, oleuropein, and apigenin-7-o-glucoside; luteolin, quercetin, luteolin glucoside, oleuropein aglycon, rutin, and luteolin diglucoside. ESI-TOF-MS can provide superior mass resolution and accuracy, whereas ESI-IT-MS/MS is suitable for obtaining fragment ions of structural relevance for identifying target compounds in a complex extract [63].

### 3.3. High-Performance Liquid Chromatography-Diode Array Detection-Mass Spectrometry (HPLC-DAD-MS)

Hyphenated techniques, particularly HPLC coupled to DAD and MS, have proven to be extraordinarily beneficial for optimizing the extraction of natural products [64]. Using a reliable HPLC–DAD–ESI-MS/MS method, 25 compounds in the extract of *Aloe barbadensis* Miller were characterized based on their spectral data or comparison with the reference standards. It was also used to check the quality of *A. barbadensis* extracts by measuring the amount of 8-C-glucosyl-7-O-methyl-(*S*)-aloesol, aloe-emodin, aloin A, and aloenin B [64]. For multi-response optimization of phenolic antioxidants from white tea (*Camellia sinensis* L. Kuntze), the contents of catechin and epicatechin in the extracts were determined by HPLC-DAD, and the phenolic profile of the extracts was identified by HPLC–DAD–Q-TOF–MS/MS in the optimal condition on the basis of their HPLC retention time, detection wavelength, and mass spectra based on the values of mass-to-charge ratio (*m*/*z*) and comparisons with fragmentation patterns. White tea extract is a good source of antioxidants, particularly flavan-3-ols, for dietary supplements, according to the results [65]. Modern hyphenated techniques can be used to optimize the extraction of natural products because they offer both high separation efficiency and the online acquisition of complementary spectroscopic data on an HPLC peak of interest within a complex extract.

### 3.4. High-Performance Liquid Chromatography-Nuclear Magnetic Resonance (HPLC-NMR)

HPLC-NMR has improved the ability to isolate and interpret the structures of complex natural products. The structural information derived from a hybrid HPLC-NMR technique may be adequate for identifying unidentified extract constituents. In general, reversed-phase HPLC columns with binary or tertiary solvent mixtures yield insufficient NMR spectra due to the presence of strong solvent signals and weak natural product signals on the same spectra. To suppress solvent signals, pre-saturation, soft-pulse multiple irradiations, or water suppression enhancement can be utilized [66]. The development of efficient solvent suppression techniques enables the measurement of reversed-phase HPLC-proton NMR spectra [67]. 2D total correlation spectroscopy (2D-TOCSY) permits the determination of all spin system neighbors simultaneously. So long as there is coupling between each intervening proton, correlations between distant protons will be observed. It can provide information on four to five bonds if each successive proton is coupled. As a result of the nuclear overhauser enhancement (NOE) effect, dipole-dipole-coupled protons in a 2D nuclear overhauser enhancement spectroscopy (2D-NOESY) experiment exchange magnetization through space. It has been successfully applied to the study of hydrogen bonds and to determining the relative configuration of natural products [42,68]. The introduction of solvent suppression strategies and their combination with homo- and heteronuclear 2D NMR experiments, such as 2D-TOCSY and 2D-NOESY, has significantly increased the potential of HPLC-NMR for the investigation and structural elucidation of unidentified natural products [69]. 

Trends and applications of various modes of HPLC-NMR operation, such as online-flow mode, stop-flow mode, and loop-storage mode, were discussed, as well as practical applications in natural product analysis. In online-flow mode, the HPLC is directly coupled to the NMR probe, and spectra are continually obtained while peaks of natural products are eluting. The NMR flow cell’s short exposure time for eluted peaks reduces its sensitivity. Depending on solvent properties, solvent composition changes during elution may alter sample and solvent chemical shifts [70]. In stopped-flow mode, the NMR probe’s interface is directly connected to the HPLC detector’s outflow. It has a higher signal-to-noise ratio and enables the detection of only particular peaks. As soon as the eluted peaks reach the NMR detection device, the HPLC pump must be halted until the NMR signals are gathered. The primary disadvantage of this mode may be its dependence on separations resolved for retention times longer than 2 min [71]. In loop-storage mode, the HPLC detector outlet must be directly connected to the sample storage loops. In these loops, the eluted peak is immediately collected. Upon completion of the separation, the HPLC pump can be used to force the previously collected peaks into the NMR flow cell using a valve. In the new offline mode, solid-phase extraction/cartridge storage mode, also known as LC-SPE-NMR, nondeuterated solvents are utilized in the HPLC system, and the separated peaks are stored in SPE cartridges. After the natural products in the SPE cartridges have been nitrogen-dried, the deuterated solvent is used to propel them to the NMR flow cell. Consequently, loop/cartridge storage provides a higher peak resolution than direct stop-flow mode [71]. Despite the fact that the loop/cartridge-storage mode eliminates the need for expensive deuterated solvents during the NMR experiment, sample degradation or structural change may occur during storage, and special pumping equipment is required to elute samples from temporary storage [72].

According to RSM-based extraction optimization for *Azadirachta indica* leaves, proton NMR-based metabolite profiling of the obtained extract revealed the presence of numerous potential bioactive compounds that can be considered a source of α-glucosidase inhibitors and antioxidants; therefore, it can also be used as active constituents of functional foods [73]. Metabolomics techniques rely on identifying as many small molecules as possible and have been used to characterize the relationships between the metabolome (the complete set of small natural molecules) and the corresponding genetic makeup, source, quality, or other biological characteristics. NMR is the primary analytical technique currently employed for metabolomics research. The optimal extraction of anthelmintic metabolites from *Lysiloma latisiliquum* leaves was optimized by comparing eight distinct solvent systems. As an internal standard, trimethylsilylpropanoic acid was used to examine the proton-NMR spectra of tannin-free extracts in methanol-d_4_. The principal component analysis (PCA) of the proton NMR data indicated hydrophilic solvents as optimal for the extraction of *L. latisiliquum* leaves with potent anthelmintic activity and revealed that the bioactive metabolites are high-polarity, glycosylated substances. The results of this study support proton-NMR metabolomics as a useful technique for the elucidation of bioactive metabolites in plant extracts in the absence of prior phytochemical studies [74]. *Croton membranaceus* root-bark extracts were analyzed using the high-performance liquid chromatography-solid phase extraction-nuclear magnetic resonance (HPLC-SPE-NMR) technique. After post-column dilution of the eluate with water, the separated peaks were trapped on SPE cartridges and eluted with acetonitrile-d_3_ into an NMR probe. Scopoletin, the main extract ingredient, was trapped more efficiently on an SPE phase (polystyrene-type polymer) than on a C18 phase. After repeated trappings, SPE cartridges have a minimum four-fold higher NMR signal-to-noise ratio than C18 cartridges. The repeated peak capture in the HPLC-SPE-NMR method permitted the acquisition of high-quality proton-detected 2D NMR spectra without solvent suppression. It was shown that excessively long T1 relaxation times may compromise experiments in which acetonitrile-d_3_ is used as the cartridge eluent. Nevertheless, the sensitivity gain provided by the HPLC-SPE-NMR technique through repeated peak trappings allowed the acquisition of good-quality proton-detected 2D NMR spectra without requiring solvent suppression [75].

### 3.5. High-Performance Liquid Chromatography-Diode Array Detection-Solid Phase Extraction-Nuclear Magnetic Resonance (HPLC-DAD-SPE-NMR)

For identifying radical-scavenging compounds in *Rhaponticum carthamoides* extracts, a high-performance liquid chromatography apparatus coupled to a solid-phase extraction unit and NMR detector (LC-DAD-SPE-NMR) was used, along with online radical-scavenging detection. Without the need for offline chromatographic stages, the technique enabled the detection and identification of particular compounds. UV spectra, 1D and 2D proton and carbon-13 NMR spectra, and MS spectra were used to reveal the structures of flavonoid glycosides. The SPE apparatus was adaptable, enabling analyte focusing, and multi-trapping. This performs better than HPLC-NMR with partially deuterated liquids. Future integration of LC-DAD-SPE-NMR with online bioassays and online MS will accelerate high-throughput screening identification and dereplication [76]. Four flavonol glycosides and three 5α-cardenolides of *Kanahia laniflora* extract were separated and identified using the HPLC-DAD-SPE-NMR technique, and their identities were subsequently confirmed by HPLC-MS data. The incorporation of online SPE in HPLC-NMR has significantly increased the sensitivity by concentrating natural products in a tiny-volume NMR flow cell and increasing the quantity of these compounds through multiple peak trapping. The HPLC-DAD-SPE-NMR technique considerably accelerates the dereplication of complex extracts by characterizing extract components [77].

### 3.6. High-Performance Liquid Chromatography-Photodiode Array Detection-Mass Spectrometry-Solid-Phase Extraction-Nuclear Magnetic Resonance (HPLC-PDA-MS-SPE-NMR)

A high-performance liquid chromatography-photodiode array detection-mass spectrometry-solid-phase extraction-nuclear magnetic resonance (HPLC-PDA-MS-SPE-NMR) could be used to directly determine the structures of unrefined extracts containing minute amounts of natural products. The results demonstrated that post-column SPE is an effective method of analyte concentration and accumulation not only for NMR but also for circular dichroism (CD) spectroscopy. Thus, the combination of the hyphenated technique and CD enabled rapid detection of (*R*)-(-)-gossypol in *Thespesia danis* twigs. Consequently, an HPLC-PDA-MS-SPE-NMR-CD system is envisioned as an efficient natural product discovery instrument, as the chirality of the compounds is a crucial property in terms of biological activity [78].

### 3.7. Gas Chromatography-Flame Ionization Detection (GC-FID)

GC-FID is characterized by its simplicity, dependability, relatively high sensitivity, and excellent linearity for a broad range of natural products. It consists of four main parts: a carrier gas source, a sample introduction system, a GC column, and a flame ionization detector (FID). FID uses the flame that results from the combustion of hydrogen and air. When compounds are introduced to a flame via the FID jet, a large number of ions are produced. A polarizing voltage is applied to the FID collector, which will attract the ions and produce a current proportional to the amount of analyte in the flame. The carrier, hydrogen, and oxygen flows must be properly adjusted for optimal FID operation [79].

GC-FID was utilized for the simultaneous quantitative analysis and chemical characterization of volatile oils extracted from *Alpinia oxyphylla* fruits by hydrodistillation. According to the results, the volatile oils contained *p*-cymene and nootkatone as significant components. A GC-FID fingerprinting method was developed, and chemometrics was used to analyze the profiles. The profiles of the vast majority of samples were uniform and stable. These findings demonstrated that GC-FID determination and fingerprinting analysis are highly effective, organized, and practicable methods for the thorough profiling of volatile oils in *A. oxyphylla* [80]. Using GC-FID, the volatile oils of 12 species of *Eucalyptus* were identified and quantified in terms of their constituents and relative concentrations. Monoterpenes, α-pinene and 1,8-cineol were the most abundant secondary metabolites isolated from *Eucalyptus globulus*. Large quantities of 1,8-cineol are also detected in *Eucalyptus mycrocoris*, *Eucalyptus resinifera*, and *Eucalyptus urophylla*. However, the chromatographic profiles of these species were significantly distinct. In conclusion, the validated GC-FID method can accurately quantify the 1,8-cineol content in various *E. globulus* matrices and aid in monitoring the entire manufacturing process. It can also be used to obtain chromatographic fingerprinting of various *Eucalyptus* species, allowing for early identification of the correct species to be used and preventing the misapplication of other closely related species [81].

### 3.8. Gas Chromatography-Mass Spectrometry (GC-MS)

Due to its simplicity of use, high sensitivity, and ability to effectively separate extracts, GC is one of the most prevalent chromatographic techniques for separating volatile substances. In gas-liquid chromatography (GLC), the mobile phase is a carrier gas, and the stationary phase is a liquid with a high boiling point that is adsorbed on an inert solid support. Since GLC is the most versatile and selective gas chromatography separation technique, the majority of natural product studies will concentrate on GLC. Components of the extract are distributed between a stationary phase and a gaseous mobile phase that transports the components through the stationary phase. The affinity of each compound with the stationary phase varies due to their diverse properties and structures. Consequently, under the same propelling force, the retention duration of various components in the column differs, resulting in their movement out of the column in various orders [40]. GC-MS is a technique that combines the characteristics of GC and MS in order to separate and identify volatile substances within an extract. Instruments such as quadrupole, ion trap, and time-of-flight (TOF) mass analyzers are widely used today. Quadrupoles are the most common mass analyzers because they can accommodate a broad *m*/*z* range and are relatively inexpensive. High-resolution time-of-flight mass spectrometry (HRMS) is exceptional in its capacity for accurate mass measurement of high-resolution fragment ions [82]. On the basis of the interpretation of fragmentations, GC-MS-obtained MS spectra provide valuable structural data. The fragment ions with varying relative abundances can be compared to spectra from a library. GC-MS is an efficient method for analyzing compounds that are sufficiently volatile, tiny, and stable at elevated temperatures. For GC-MS analysis, polar compounds, particularly natural products with multiple hydroxyl groups, may require suitable derivatization. Typically, derivatization is performed to modify the analyte’s properties for improved separation and to increase the method’s sensitivity. Additionally, it may enhance the capacity for identification. Alkylation, the formation of aryl derivatives, silylation, acylation, and several other forms of derivatization are more frequently employed in analytical applications. The most common method for the derivatization of natural products is silylation (e.g., formation of trimethylsilyl derivatives), which involves transforming the analyte into its trimethylsilyl derivative [83].

Betel leaf (*Piper betle* L.) var. *Bangla* is a volatile, oil-rich plant belonging to the *Piperaceae* family that is traditionally employed as an herbal medication. The optimization of volatile oil extraction from betel leaves and biochemical characterization is of great interest for industrial applications. The GC-MS analysis uncovered numerous volatile compounds with a variety of antimicrobial activities. Different percentages of eugenol, estragole, linalool, α-copaene, anethole, chavicol, and caryophyllene were discovered to be abundant in the betel leaf extract [84]. Using GC/GC-MS, the plant *Aethionema sancakense* was identified as a new species based on its essential oil and fatty acid compositions. Linoleic acid, α-humulene, camphene, and heptanal were identified as the principal essential oil components of the aerial part of *A. sancakense* [85]. Typically, the volatile profile is determined by headspace solid phase microextraction (HS-SPME), followed by GC analysis with mass spectrometric detection. The use of HS-SPME GC-MS for fingerprinting and the identification and quantification of specific classes of natural products is quite prevalent [86]. It was used to analyze samples of organic commercial multi-flower honey in order to identify biological biomarkers such as carboxylic acids, alcohols, aldehydes, ketones, terpenes, esters, norisoprenoid, and furan derivatives. [87]. 

Currently, the identification of natural products by multiple hyphenations has become an established method for determining the structures of the constituents present in complex extracts. The hyphenation of HPLC or GC coupled to DAD, MS, SPE, or NMR is an effective analytical tool for rapid and robust dereplication of complex extracts, enabling simple identification of known compounds as well as comprehensive structure elucidation of novel compounds directly from complex extracts.

## 4. Quality Control, Standardization and Biological Activity of Extracts

Natural products have distinct characteristics compared to conventional synthetic molecules. They are distinguished by their structural diversity and complexity. In general, natural substances have a larger chemical structure, a greater number of sp^3^ carbon and oxygen, fewer nitrogen and halogen, a greater number of hydrogen bond acceptors and donors, higher hydrophilicity (a lower n-octanol-water partition ratio), and greater molecular rigidity than those of synthetic compounds [88,89]. Although the selection of raw materials of plants and animals for study is sometimes based on their traditional medicinal uses, this is not always the case; other characteristics, such as chemical diversity and the absence of previous studies, may also justify further study. Numerous natural products have been reported to possess a variety of intriguing and important biological activities, including antioxidant, antimicrobial, antiparasitic, anti-inflammatory, anti-diabetic, and antiproliferative [90]. Plants are a rich source of phytochemicals with a variety of health benefits, including protection from cancer, atherosclerosis, cardiovascular disease, diabetes, and obesity [91,92,93]. Therefore, extracting and identifying bioactive natural products is of considerable interest. Typically, a crude extract’s biological activity is evaluated in vitro. Using in vitro assays, the potential of natural product constituents as significant anti-disease and health-enhancing compounds is demonstrated [94].

Natural products for human sustenance are typically extracted using aqueous ethanol, aqueous glycerin, or hot water as extracting solvents. Due to their respective boiling points, removing ethanol is difficult, removing water is more difficult, and removing glycerin is the most difficult. In addition, water extracts are inherently unstable and contain polar components that impede chemical analysis [95]. Standardized extracts or products contain specific amounts of chemical constituents. Standardization involving multiple analytical methods, including hyphenated techniques, provides information that could be beneficial for selecting and adjusting dosages [96,97]. Standardized extracts may contain fewer chemical constituents than non-standardized extracts, as the process of increasing concentrations of some constituents may entail extraction techniques that reduce extract complexity. Reduced extract complexity can be disadvantageous if the actual bioactive constituent is unknown, as standardization of certain constituents may result in lower dosages of bioactive constituents responsible for biological effects [98]. The concentrations of active or marker metabolites in extracts have been determined and conform to the pharmacopoeia monograph. 

Natural product extracts must contain various contaminants, including heavy metals, pesticides, residual solvents, fungal spores, toxins, or pathogens, within permissible limits [98]. Certain contaminants can only be detected using specialized analytical procedures. GC-MS, for example, is suitable for the analysis of particular pesticides and residual solvents, but it is insensitive to a large number of non-volatile contaminants [99]. In order to detect heavy metal contamination, an inductively coupled plasma-mass spectrometer (ICP-MS) must be utilized. Consequently, it should carefully consider which analytical techniques are suitable for detecting specific contaminants [100].

Natural products remain a promising resource for the discovery of scaffolds with high structural diversity and diverse bioactivities that can be optimized for extraction and developed into novel formulations. However, the technological advancements discussed in this article provide a firm foundation for natural product extracts to continue producing significant health and longevity-related products.

## 5. Examples of the Extraction and Chemical Analysis of Natural Products

Important sources for the prevention and treatment of human diseases are natural products. Because the concentrations of bioactive natural products in extracts are typically quite low, it is crucial to devise efficient and selective methods for their extraction. This article intends to provide examples of natural product extraction and chemical analysis.

### 5.1. Extraction and Chemical Analysis of Plant Extracts

Baby corn silk is the stigma and style of unfertilized maize (*Zea mays* Linn.) female blossoms. Individually dried silk was macerated in 40% *v*/*v* ethanol and digested in distilled water. The phytochemical constituents of both obtained extracts were determined using color reactions, TLC screening, UV-visible, FTIR, and proton NMR experiments. Baby corn silk extracts were found to contain flavonoids, tannins, terpenoids, and steroids. In addition, the 40% *v*/*v* ethanol extract had a substantially higher total phenolic and flavonoid content and significantly stronger antioxidant activity than the aqueous extract [101]. 

Anti-inflammatory, antimicrobial, antiviral, and antiprotozoal properties were present in coconut kernel extracts. Therefore, it was crucial to investigate the optimal coconut kernel extraction techniques in order to obtain the highest concentration of active metabolites and the strongest bioactivity. Under various conditions, coconut kernels were extracted, including maceration with 95% *v*/*v* ethanol, maceration with 50% *v*/*v* ethanol, and soaking in heated distilled water. TLC, FTIR, and proton NMR analyses revealed that all extracts contained trilaurin, terpenoids, flavonoids, and carbohydrates; however, the extract obtained by soaking in boiling water demonstrated the highest antioxidant activity and total phenolic content [102]. Results indicated that the polar antioxidants in coconut kernels dissolved effectively in water. 

The antioxidant and anti-inflammatory properties of *Andrographis paniculata* and *Tinospora crispa* extracts were correlated with their phenolic and other phytochemical components [103,104]. *A. paniculata* and *T. crispa* extracts could potentially be used as bittering agents in anti-nail-biting lacquers due to their bitter taste and biological activities. Despite having a substantially higher total phenolic content and antioxidant activity, *T. crispa* extract was significantly less bitter than *A. paniculata* extract [105]. The results demonstrated that bittering compounds and antioxidants could be extracted from both plants using 95% *v*/*v* ethanol. The FTIR analysis of extracts of *A. paniculata* and *T. crispa* confirmed the presence of acrid compounds, including andrographolides (terpenoids) and aporphine alkaloids, respectively. 

Due to their rich rhein and phenolic compositions, *Senna alata* leaves exhibit a wide range of biological activities. The utilization of modern extraction techniques in combination with experimental design has led to the preparation of higher-quality extracts compared to traditional extraction methods lacking experimental design. A comparative example between fermentation, a traditional method, and UAE, a modern method, demonstrates this improvement [106,107]. While the extraction yields obtained from fermentation and UAE were similar, the UAE extract displayed higher levels of bioactive substances such as total phenolic content and rhein content when compared to the extract from fermentation. However, through the optimization of extraction conditions using experimental design, the total phenolic content, and rhein content in the UAE extract can be significantly increased. Furthermore, the optimized UAE extract exhibited a stronger antioxidant effect than the extract obtained from fermentation. Based on these experimental results, it can be concluded that the combination of modern extraction techniques and experimental design yields extracts with higher quantities of active substances and enhanced biological activity compared to traditional extraction methods. 

Through the utilization of an experimental design, the bioactive de-chlorophyll rhein-rich *S. alata* extract was prepared employing the UAE method and LLE with coconut oil. The primary objectives were to enhance the rhein content and diminish the dark color caused by chlorophyll. The rhein content was accurately identified and quantified using a validated HPLC-DAD method. The presence of rhein and phenolics in the de-chlorophyll extract potentially contributes to its antioxidant, anti-inflammatory, and antibacterial properties. These findings strongly indicate that the de-chlorophyll rhein-rich *S. alata* extract, produced through the optimized UAE coupled with LLE technique, exhibits considerable biological activity, making it a promising candidate for the development of cosmeceuticals and pharmaceuticals [107]. 

As demonstrated by the aforementioned studies, in order to extract biologically active substances from plant materials, the extraction method and type of solvent must be considered. In addition, phytochemical constituents and biomarker contents can be identified using spectroscopic methods and hyphenated techniques, respectively.

### 5.2. Extraction and Chemical Analysis of Extracts from Animal Tissues

Male deer of the *Cervidae* family have valuable velvet antlers on their frontal protuberances. Asians consumed extracts of velvet antlers for their antioxidant, antifatigue, immunomodulating, and libido-enhancing properties because they contained bioactive compounds with low toxicity, such as testosterone, chondroitin sulfate, phospholipids, p-hydroxybenzaldehyde, and protein hydrolysate. Because velvet antlers are valuable natural raw materials, the UAE used a sequential multi-solvent method to extract bioactive components from velvet antler samples. The chemical constituents of the obtained hexane extract, 75% *v*/*v* ethanol extract, and water extract were determined using TLC, FTIR, proton NMR, and HPLC-UV analysis. Oleic acid, linoleic acid, α-linolenic acid, and testosterone were detected in hexane extract and 75% *v*/*v* ethanol extract. A total of 75% *v*/*v* ethanol extract exhibited the highest concentration of testosterone and the most powerful antioxidant activity. All of the extracts contained essential elements, but their levels of toxic elements and microbial contaminants were below ASEAN’s acceptable threshold. The 75% *v*/*v* ethanol extract was regarded as a safe and effective source of antioxidants and testosterone [108].

Beef tallow can be used to treat skin disorders including aging skin, dermatitis, and psoriasis, due to its fatty acid composition and antioxidant activity. To generate beef tallow, soft fat and hard fat from raw beef adipose tissues were extracted using various rendering methods, including low-temperature, double-boiling, and MAE methods. All of the physicochemical and quality characteristics of tallow samples derived from various rendering methods fell within the permissible range. The GC-FID method was utilized to determine the fatty acid contents of tallow samples. The results revealed that tallow prepared from soft fat using low-temperature rendering was the lightest yellow, had the highest antioxidant activity, and contained the highest concentration of unsaturated fatty acids, including palmitoleic acid, oleic acid, linoleic acid, and linolenic acid. Therefore, rendering at a low temperature should be the most effective extraction method. The MAE method produced tallow with the greatest yield and the lowest antioxidant activity [109]. Increased microwave irradiation power altered the polarity, viscosity, and surface tension of the extraction solvent, resulting in improved extraction efficiency [110]. The MAE method employing microwave-based apparatus is related to the oscillating electric field that induces molecular frictions of dipole water molecules, resulting in a rapid increase in adipose tissue temperature that induces undesirable degradation reactions [110,111]. In accordance with prior research [112], these results suggest that the MAE method’s high extraction temperature leads to the degradation of antioxidants despite its high extraction efficiency. This demonstrates that modern extraction methods have the potential to enhance the extract yield. However, inadequate control over extraction conditions can lead to the degradation of active bioactive compounds.

Ostrich oil has become recognized for its nutritional, cosmetic, and pharmaceutical purposes, due to its high concentration of essential fatty acids. The quality of ostrich oil is determined by its polyunsaturated fatty acid (PUFA) content and antioxidant activity. Two methods were used to extract ostrich oil from abdominal adipose tissue: the conventional method with a higher temperature and a newly developed method with a lower temperature. The developed technique produced ostrich oil with a higher total PUFA content and greater antioxidant power than the conventional method [113]. A high extraction temperature could destroy PUFAs and reduce antioxidant activity, according to the results.

Animal tissue extracts frequently contain proteins, fatty acids, hormones, and enzymes that are sensitive to heat and light. Therefore, an extraction procedure that does not involve extremely high temperatures must be selected. Due to the volatility of fatty acids, GC is typically used to analyze them. 

As mentioned previously, plant and animal extracts can be utilized as dietary supplements and cosmetic ingredients due to their diverse biological activities.

## 6. Electrospinning

Several techniques, such as template synthesis, phase separation, self-assembly, and electrospinning, can be utilized to create nanofibers. Electrospinning is a technology that creates ultrafine continuous fibers with multiple applications. Electrospinning’s significant technological advancement enables the design and synthesis of novel polymer materials with ideal properties. Electrospun fibers have numerous applications in the medical field, including the delivery of therapeutically active compounds, tissue engineering, biosensors, and wound dressing [114].

### 6.1. Principles of the Electrospinning

**Figure 1:**
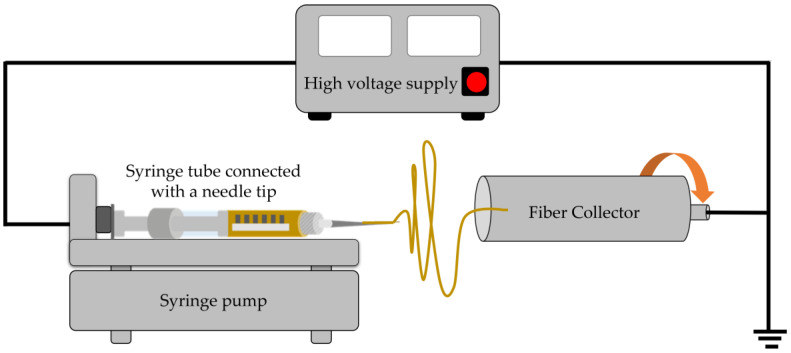
Schematic of typical instrumentation for electrospun fibers: a high-voltage supply, an injection part consisting of a syringe pump and a syringe tube connected with a needle tip, and a fiber collector.

Electrospinning is an electrohydrodynamic process involving the electrical charging of droplets to produce a jet, which is then stretched and dried to produce fibers. As shown in Figure 1, a typical electrospinning system comprises a high-voltage DC or AC power supply, an injection pump, and a fiber collection device [115]. In general, the electrospinning procedure consists of four steps: (1) the droplet is charged and forms a Taylor cone or conical jet; (2) the jet, consisting of droplets with the same charge, is derived and elongated along a straight line; (3) the jet bends from a straight line to all sides and beats continuously; and (4) the fibers are solidified by evaporation of the jet solvent and collected on a grounded collector [116].

In electrospinning, the polymer solution or melted polymer (with or without active compounds) is fed into a syringe and pumped through a needle that is connected to a high-voltage power supply (usually between 1 and 30 kV). When a high voltage is applied, an electric field is generated between the tip of the needle and the collector. Due to the high-voltage supply and surface tension, the polymer solution or melted polymer is ejected from the spinneret (typically a syringe needle) to create droplets during the electrospinning process. When the electrical charges or electrostatic repulsion of a polymer solution are greater than the surface tension, polymer jets in the shape of a Taylor cone are formed. Typically, polymer jets are initially stretched in a straight pattern and then transformed into a vigorously non-directional line due to the jets’ instability. The polymer jets are then solidified and deposited on the collector, which is typically covered with aluminum foil [117].

Electrospinning is a cost-effective technology that can be easily adapted from laboratory research to industrial production. Additionally, this technology can be utilized to produce nanofiber structures with a high specific surface area and remarkable inter- and intra-fiber porosity. In addition, it allows the mixing of two or more active ingredients and may facilitate or inhibit the burst release of active compounds, as well as the achievement of modified release. Electrospinning is capable of spinning a wide variety of natural and synthetic polymers with tunable surface morphology and interconnected porosity with variable pore size [115,116].

Important aspects of solution electrospinning [118] include:

- Appropriate solvents for dissolving the polymer should be available. The viscosity and surface tension of the solvent should not be too high to prevent the formation of a jet, nor should they be too low to allow the polymer solution to flow freely from the nozzle.
- The applied voltage must be sufficient to overcome the viscosity and surface tension of the polymer solution in order to create and maintain a jet from the syringe tip.
- The length between the spinneret and the grounded surface should be sufficient for solvent evaporation in time for fiber formation, but not so narrow as to produce sparks between the electrodes.

Compared to other nanostructures, electrospun nanofibers exhibit unique properties. The following are some benefits of electrospun fibers [115,119]:

- Electrospun nanofibers with their high porosity and similarity to the natural extracellular matrix (ECM) can promote cell adhesion, proliferation, migration, and differentiation.
- A high specific surface area is advantageous for wound exudate, the dispersion of bioactive compounds, and enhancing the solubility of substances that are poorly water-soluble.
- Fiber morphology is advantageously utilized as a multifunctional wound dressing material.
- Enhancement of bioactive compound encapsulation effectiveness.
- Variable surface architecture.
- Decreased burst release and the potential for efficient surface functionalization through the appropriate selection of a bioactive compound-polymer-solvent system.

### 6.2. Electrospinnable Polymers

The majority of electrospun fibers consist of natural biopolymers, such as silk fibroin, chitosan, and collagen. Various types of electrospun fibers are produced using synthetic polymers, including polyvinyl alcohol (PVA), polyvinyl chloride (PVC), and polylactic-co-glycolic acid (PLGA). In addition, the combination of certain polymers (such as chitosan-PVA and chitosan-silk fibroin) can be used to produce the desired fibers [120,121].

#### 6.2.1. Natural Polymers

Several natural polymers exhibit biodegradability, biocompatibility, and nontoxicity. Electrospun fibers made from natural polymers have attracted the attention of researchers because they can stimulate the natural cells in the human body for a higher level of functionality and form a fibrous scaffold that mimics the ECM to support cell activities [122]. In addition, natural polymers contain a variety of functional groups, which facilitate the modification of their structures. However, it has been discovered that the lack of stability in physiological conditions and poor mechanical properties are the main obstacles when natural polymers are used [123].

#### 6.2.2. Synthetic Polymers

Recently, the most common synthetic polymers, such as polylactic acid (PLA), polyethylene oxide (PEO), PVC, and PVA, have replaced natural polymers in the production of electrospun fibers due to their versatility in applications and superior mechanical properties. Combining these polymers with other synthetic and natural polymers can improve their mechanical and physical properties and facilitate controlled release [124].

#### 6.2.3. Blend Polymers

Combining natural polymers is a straightforward method for producing substrates suitable for cell activities. However, their primary drawbacks include rapid biodegradability, poor processability, and poor mechanical properties. Combining natural and synthetic polymers is an additional method for producing electrospun fibers, as blend polymers can be fabricated into a variety of porous structures and provide optimal support for cell attachment and proliferation [125].

### 6.3. Development of Shellac-Based Electrospun Fibers

Because of the mechanical characteristics, controlled biodegradation, and biological compatibility of shellac, electrospun fibers made from shellac, a natural polymer derived from the insect *Kerria lacca*’s secretion, appear to be gaining widespread popularity [126,127,128,129,130,131,132]. Consequently, this article explores the creation of bioactive shellac electrospun fibers. Shellac resin is a complex mixture of polyesters and single esters with hydroxyl and carboxyl groups that primarily consists of jalaric and laccijalaric acid derivatives [133]. It is a semi-crystalline polymer with low crystallinity, a melting point between 50 and 75 °C, and solubility in alcohols (ethanol and isopropyl alcohol) and alkaline solutions (pH above 7.4) [133,134,135]. By refining sticklac harvested from lac-bearing branches, various types of shellac can be produced. In one process, seedlac is dissolved in an aqueous alkaline solution, then treated with sodium hypochlorite, and precipitated with sulfuric acid. The precipitate is then washed with water and dried to produce bleached, dewaxed shellac [136]. Several studies have been conducted on the application of certain types of shellac for the electrospinning fabrication of nanofibers [128,130,137]. In general, electrospinning of natural polymers is difficult due to the behavior of polymer solutions and the specific conditions required to form solutions and maintain a liquid state. Nevertheless, shellac has an advantage in electrospinning due to its solubility and stability in anhydrous ethanol, as well as its appropriate molecular weight [130,136].

Wang, X., et al. investigated the coaxial electrospinning fabrication of medicated shellac nanofibers with colon-specific sustained release. The core fluid was a mixture of 75% *w*/*v* shellac and 15% *w*/*v* ferulic acid in ethanol, while the shell was a mixture of ethanol and N,N-dimethylformamide (8/10 *v*/*v*). The results of X-ray diffraction and differential scanning calorimetry revealed that ferulic acid was distributed amorphously in the fibers. IR spectra also showed that ferulic acid and shellac have hydrogen bonds between them. Due to the presence of hydroxyl and carbonyl groups in both ferulic acid and shellac, the characteristic peaks of ferulic acid at 1689, 1663, and 1619 cm^−1^ due to the vibration of carbonyl groups appeared as a single peak at 1698 cm^−1^ in the spectra of fibers. In addition, numerous peaks in the fingerprint region of the ferulic acid spectrum have vanished from the spectra of the fibers. These observations demonstrate that drug molecules combine with shellac through the formation of hydrogen bonds. The ferulic acid release was minimal at pH 2.0 and sustained in a neutral dissolution medium, according to in vitro dissolution tests [126].

Chinatangkul, N., et al. investigated, utilizing a full factorial design, the fabrication factors influencing the formation and properties of shellac nanofibers loaded with an antimicrobial agent, monolaurin [128]. On nanofiber characteristics, the effects of formulation and process parameters including shellac content (35–40% *w*/*w*), monolaurin content (1–3% *w*/*w*), applied voltage (9–27 kV), and flow rate (0.4–1.2 mL/h) were investigated. The shellac content was the most influential factor on fiber diameter and had a negative effect on bead formation, according to the results. Using the response surface area at an applied voltage of 18 kV and a flow rate of 0.8 mL/h, small (488 nm) and beadless (0.48 beads per fiber) fibers with a shellac-to-monolaurin weight ratio of (37.5:1.1) were produced. Due to the hydrophilic structure of its outer membrane, shellac nanofibers loaded with monolaurin exhibited excellent antibacterial activity against *Staphylococcus aureus* in a time-kill kinetics assay. Shellac nanofibers containing monolaurin have the potential to be used as a medicated wound dressing.

Electrospun shellac fibers were developed in order to transport an extract of *K. parviflora* rhizomes containing bioactive methoxyflavones. Using a Box–Behnken design, the optimal production parameters that influence the fiber diameter and bead-to-fiber ratio responses were determined. By combining 37.25% *w*/*w* shellac and 1.50% *w*/*w* rhizome extract with a solution feed rate of 0.8 mL/h and an electrical voltage of 18 kV, the optimization conditions produced fibers with a small diameter (574 nm) and a lower bead-to-fiber ratio (0.48 beads per fiber). Throughout the electrospun shellac fibers, the extract was detected during the characterization study. The results were highly correlated with their theoretical counterparts, suggesting that the regression models used to predict the response variables were accurate. An in vitro dissolution study demonstrated that *K. parviflora* rhizome extract-loaded electrospun shellac fibers are capable of producing a sustained-release profile within 10 h [137]. Due to the antimicrobial, anti-inflammatory, and antioxidant properties of *K. parviflora* rhizome extract, shellac fibers electrospun with this extract could be developed as wound dressings.

The morphology of electrospun nanofibers is important in determining their final physical and chemical properties, which are primarily governed by electrospinning. The influential factors affecting fiber morphology in the electrospinning process can be categorized into three main types: process parameters, solution parameters, and ambient conditions. The morphology and structure of nanofibers can be controlled by adjusting these parameters. Therefore, the appropriate conditions of electrospinning should be scrutinized to obtain the desired range of beadless and ultrafine nanofibers. 

The morphology of electrospun fibers plays a crucial role in determining their ultimate physical and chemical properties. Process parameters, solution parameters, and ambient conditions are the three primary types of influential factors that affect fiber morphology in electrospinning [138]. By adjusting these parameters, the morphology and structure of fibers may be altered. In order to obtain the desired range of beadless and small-diameter fibers, it is necessary to investigate the optimal electrospinning conditions, which will be discussed in the next section.

### 6.4. Parameters Affecting the Quality of Electrospun Fibers

Although the electrospinning method is versatile and straightforward, it may be affected by a number of parameters [139]. Optimizing the electrospinning parameters is necessary for producing nanofibers with the desired morphology, structure, and properties. Polymer solution parameters (e.g., viscosity, surface tension, and electrical conductivity); processing parameters (e.g., applied electric voltage, solution feed rate, and distance between the needle tip and collector); and environmental parameters (e.g., relative humidity and temperature) influence the electrospinning process [140].

#### 6.4.1. Polymer Solution Parameters

- Viscosity

Polymer concentration has a significant impact on the viscosity of electrospun solutions. In addition, the temperature may affect this parameter. Due to a higher level of polymer chain entanglement (interaction between polymer chains), a solution’s viscosity increases, resulting in an increase in fiber diameter and the formation of smooth fiber as opposed to beaded fiber [141].

- Surface tension

In the initial step of electrospun fiber production, the electrical charge of the electrospun solution must be greater than the solution’s surface tension. Therefore, a solution with a high value of surface tension can be difficult to spin from the needle tip, necessitating the use of a strong repulsive force and resulting in the formation of beads [142]. Reducing the surface tension of the electrospun polymer has been observed to be an effective method for improving the beadless morphology of fibers.

- Electrical conductivity

The electrical conductivity of an electrospun solution has a substantial effect on the electrospinning procedure. Low-conductivity electrospun solutions cannot induce Taylor cone formation, making the electrospinning process impossible. Nonetheless, if the conductivity is too high, the polymer jet may be unstable and produce fibers with beads. Additionally, an increase in conductivity may result in a reduction in fiber diameter [143].

#### 6.4.2. Processing Parameters

- Applied electrical voltage.

An appropriate level of electrical voltage is required to initiate the formation of the fiber. The optimally applied voltage varies based on the type of polymer being utilized. A voltage that is too low cannot produce electrospun fibers, while a voltage that is too high causes an electric arc. Nanofibers with a decreasing diameter can be formed as the applied voltage is increased due to the stretching of the polymer solution in conjunction with the charge repulsion within the polymer jet. Nonetheless, a number of studies have demonstrated that increasing the applied voltage has a negligible impact on the fiber diameter [137,144].

- Solution feed rate

In the electrospinning process, the feed rate refers to the rate at which polymer solution is expelled from the spinneret to the collector. Typically, a syringe pump is used to drive polymer solution under hydrostatic pressure. The solution feed rate also has an effect on the fiber’s morphology. A rise in flow rate may result in the formation of beads as a result of insufficient drying time and an excess of polymer jet to be dried. In addition, a higher feed rate results in a larger fiber diameter. This is due to the continuous delivery of a greater quantity of polymer jets during the process [145].

- Distance between the needle tip and collector

Due to its dependence on deposition time, evaporation rate, and instability phase, the distance between the needle tip and collector can readily affect the nanofiber morphology. The optimal distance between the needle tip and the collector is 10–20 cm [142]. Charge repulsion between ions in the solution causes the polymer jet to elongate while in flight. During flight, the polymer solution solidifies as the solvent evaporates. Increasing the distance between the needle tip and collector decreases fiber diameter [146]. Nonetheless, the extremely low and high distances may lead to bead formation [147].

#### 6.4.3. Environmental Parameters

- Temperature and humidity

In addition to the electrospinning solution and processing parameters, environmental parameters, specifically temperature and humidity, can also affect the fiber’s morphology. The viscosity and solvent evaporation rate of an electrospun solution are directly affected by temperature. At elevated temperatures, viscosity decreases, resulting in the formation of a thinner fiber. Moreover, a higher temperature permits more energy to facilitate the solvent’s evaporation rate. Humidity also influences the rate of solvent evaporation. Higher levels of humidity slow down the rate of solvent evaporation, resulting in a longer time for fiber stretching. Extremely high levels of humidity can lead to the formation of a fused fiber with poor mechanical properties, especially if the polymer is hygroscopic [140].

To produce nanofibers with the desired morphology, structure, and properties, it is necessary to optimize the electrospinning parameters. Various parameters, such as the applied voltage, flow rate, viscosity, and conductivity of the electrospun solution, the distance between the needle tip and collector, the solvent used, etc., affect the properties of electrospun fibers. The parameters of electrospinning and their effects on the diameter, morphology, and structure of nanofibers are summarized in Table 3.

Electrospun fibers have gained widespread acceptance in numerous applications due to their properties, which include their high surface area, high porosity, high mass-to-volume ratio, and modifiable physicochemical properties [149,150]. Numerous variables influence the electrospinning procedure, resulting in a relatively high number of experimental runs. A method for systematically determining which variables have a significant impact on responses under controlled conditions is an experimental design. Moreover, it produces more accurate results and is more cost effective than traditional design [151]. In the order listed below, it will be discussed in greater detail.

## 7. Design of Experiment (DOE)

The design of experiments (DOE) or experimental design is a method for systematically identifying independent parameters influencing response variables, such as processability, physical properties, or product performance. DOE is a mathematical tool used to determine the significance or insignificance of particular processing and/or product variables and interactions in contributing to the measured effect, and to optimize system performance while maximizing attributes. DOE employs statistical methodology to analyze data and predict product performance under all conceivable conditions within the experimental design’s limitations, and to generate the necessary information with the least amount of experimentation. The advantage of employing a DOE strategy is that systematic data are collected, analyzed, and evaluated in order to reach objective and effective conclusions. The general structure of the DOE includes planning, designing, conducting, and analyzing experiments. The planning phase involves problem identification, response selection, and parameter estimation. The designing phase includes screening designs and optimizing designs. The conducting phase involves carrying out the experiments, and the analyzing phase involves interpreting the results and drawing valid conclusions [152]. Experiments are conducted to validate hypotheses through testing and investigation. Their purpose is to determine the variables or inputs that have a significant impact on responses or outputs, as well as identify the optimal values for those variables to achieve desired outcomes. Experimental design is widely recognized as the most effective approach for examining and optimizing significant or critical variables with the fewest number of experimental runs. Furthermore, it provides a wealth of data, including the interaction between variables on process outputs, which can be analyzed using mathematical and statistical models [153].

### 7.1. Experimental Design and Its Terminology

DOE is a method for systematically planning experiments. The purpose of this procedure is to describe the relationships between input variables and output responses. After applying this procedure, the mathematical model is obtained and then utilized to generate an acceptable design space, thereby achieving the desired results [154]. 

The following is the terminology for DOE [153]:

Factor or Parameter: A factor or parameter is an independent variable that influences the responses in an experiment. It can be controlled or uncontrolled. Uncontrolled factors are not directly under the experimenter’s control, but they may have an impact on the responses.

Response: A response, also known as a dependent variable, is the measurable outcome of interest in an experiment. It represents the variable being observed or measured to determine the effect of the factors being studied.

Level of Factor: The level of a factor refers to the assortment of values attributed to that factor. In an experiment, each factor can have multiple levels, representing the different values or conditions being tested.

Treatment: A treatment is a specific combination of factor levels that represents a particular condition in an experiment. It involves assigning predetermined values to the factors at specific levels.

### 7.2. Process of the Experimental Design

Briefly, DOE consists of three fundamental steps: the screening step (to eliminate insignificant variables), the optimizing step or response surface methodology (to determine the optimal levels for each variable), and the verification step or model validation (to confirm the optimization step and compare the predicted values to the experimental results) [153]. There are two situations in which DOE is useful. The first circumstance involves attempting to comprehend the primary governing factors and determining the optimal value for the dominant factors. Prior to optimization, it is advantageous to use a screening design in a process that saves both time and labor. When there are few factors (2–4 factors), the 2-level full factorial design can be used for screening. For screening purposes, the Plackett-Burman design (PBD) or fractional factorial design may be utilized when the number of factors exceeds five. A PBD necessitates a minimum number of experimental trials to identify influential variables. The objective of optimization studies is to identify the optimal levels of factors. Choosing the levels to be investigated is crucial for optimization. A small number of factors can be optimized using Taguchi design, central composite design (CCD), or Box-Behnken design (BBD). The BBD design provides a more economical alternative to other optimization designs [153].

#### 7.2.1. Screening Design

Prior to conducting the experiments, all variables that could affect the results should be cataloged. The number of variables is used to calculate the total number of experiment runs. The screening design is of critical importance for researchers to select the most influential variables for further optimization. The simplest method for implementing a screening design is to vary each factor individually while leaving the others unchanged. This technique is also known as one-factor-at-a-time, one-variable-at-a-time, and single-factor analysis [154]. The primary drawbacks of this design are that interactions between factors are ignored and a large number of experiments are necessary. As a result, numerous screening designs have been developed to address these deficiencies.

- Full factorial design

For a full factorial design, the response is determined for all possible factor-level combinations. *L^k^* experimental runs are required, where *L* is the number of levels and *k* is the number of factors. In the case of a full factorial design for screening approaches, a 2-level full factorial design with only 2-level factors is typically employed. The number of runs for a 2-level full factorial design is therefore 2*^k^*. A 2-level full factorial design with six factors requires 64 runs, whereas a design with nine factors requires 512 runs. As the number of factors increases, the total number of runs increases exponentially [155].

- Fractional factorial design

The primary limitation of a full factorial design is that the number of experiments increases proportionally to the number of factors and levels. Because it represents a portion of a full factorial design, a fractional factorial design is suggested to reduce the number of experiments. This design has two levels, one of which is a partial factorial design. A fractional factorial with two levels can accommodate up to eleven factors (2*^k^*^−*p*^), where *k* is the number of factors, *p* is the fraction index, and *N* is the number of experiments [156]. For example, if *k* is 8, a full factorial design would require 256 experiments. However, with *p* = 1, 2, 3, and 4, a fractional factorial design would only require 128, 64, 32, and 16 trials, respectively. Table 4 provides the number of treatments for the fractional factorial design. It is worth noting that as the number of fractions (*p*) increases, the resolution or precision of the model may diminish [157].

- Plackett-Burman design

The PBD is helpful for identifying which variables should be investigated in a subsequent optimization procedure. It functions both at low and high levels. This design estimates the linear effects of all variables for a specified sample size. It is utilized to examine *k* = *N* − 1 variables, where *k* represents the number of variables and *N* (number of experiments) is a multiple of 4 (*N* = 12, 16, 20, 24, 28, 32, 36, etc.) [156]. For example, for eleven independent variables, a total of twelve experimental runs are conducted. Due to the exclusion of unimportant factors, a large number of variables can be determined with a small number of experiments. However, this design can only be used to investigate the effects of the model’s main factors; model interactions are ignored. Utilizing a screening design, the essential factors and their interactions are evaluated. For screening experiments, 2-level full factorial design, fractional factorial design, and PBD are frequently employed [153]. Table 5 summarizes the advantages and disadvantages of various screening designs.

The resolution must be considered in order to determine whether each parameter is confounded. Resolution, which ranges from Resolution III to Resolution V, refers to the capacity of each design to estimate and evaluate the effects and interactions without confounding impacts, an effect that undermines the purpose of a process [157]. The design of Resolution III reveals that the principal effects are not confounded (or aliased) with the other principal effects. However, the two-factor interactions may confound each other. If the two-factor interaction is significant for the response variables, the results of the design with Resolution III may be misleading. The design of Resolution IV reveals that the main effects are not aliased with other main effects or two-factor interactions. Nevertheless, it can be aliased by three-factor interactions. Moreover, two-factor interactions are aliased with one another. The choice of the screening design with Resolution IV is widely accepted, as the main effects are unambiguously discernible without the aliased effects of two-factor interactions. In comparison to the full factorial design, the Resolution V (or higher) design demonstrates the design’s optimal performance. It indicates that there are no aliased main effects or two-factor interactions. However, the two-factor interaction can be aliased with three-factor interactions (or higher). In the analysis, the effects of three or more factor interactions on response variables are frequently negligible [157].

#### 7.2.2. Optimization Design

After identifying the significant variables, it is necessary to optimize the process of determining the relationship between each variable and response. Response surface methodology (RSM), a collection of useful mathematical and statistical techniques for the approximation and optimization of stochastic models, is used to investigate regression models and optimize a response (an output or response variable) that is affected by multiple independent variables (input variables or control factors) [158]. After identifying the critical variables, the remaining factors must be optimized by deriving specific experimental designs. This section discusses the optimization designs, including the BBD and the CCD.

- Box-Behnken design

In an incomplete block design, BBD contains fractional factorials with three levels per factor. *N* = 2*k* (*k* − 1) + *C_p_* was used to estimate the number of experimental runs for BBD, where *N* is the number of experimental runs, *k* is the number of factors, and *C_p_* is the number of center points. BBD is an economical design that identifies the optimal point of each factor and its interactions. This design is advantageous when it is impossible to evaluate extreme conditions (extremely low and extremely high) [159].

- Central composite design

CCD combines three levels of full or fractional factorial design with star and center points. As a result, CCD has five levels for each factor (extremely low level represented by −α, low level represented by −1, medium point represented by 0, high level represented by +1, and extremely high level represented by +α). In other words, the experimental points of this design extend beyond the maximum and minimum values of each parameter. CCD experimental runs can be calculated using the formula *N* = *k*^2^+ 2*k* + *C_p_*, where *N* represents the number of experimental runs, *k* represents the number of factors, and *C_p_* represents the center point number [160].

#### 7.2.3. Verification of an Optimization Model

After the optimization process is complete, model verification must be performed to ensure the model’s reliability under the obtained conditions. The purpose of the verification procedure is to evaluate and validate predicted models. If the predicted values are similar to the experimental values, the models can be utilized to determine and optimize the response values [152].

#### 7.2.4. Statistical Analysis for Experimental Design

**Figure 2:**
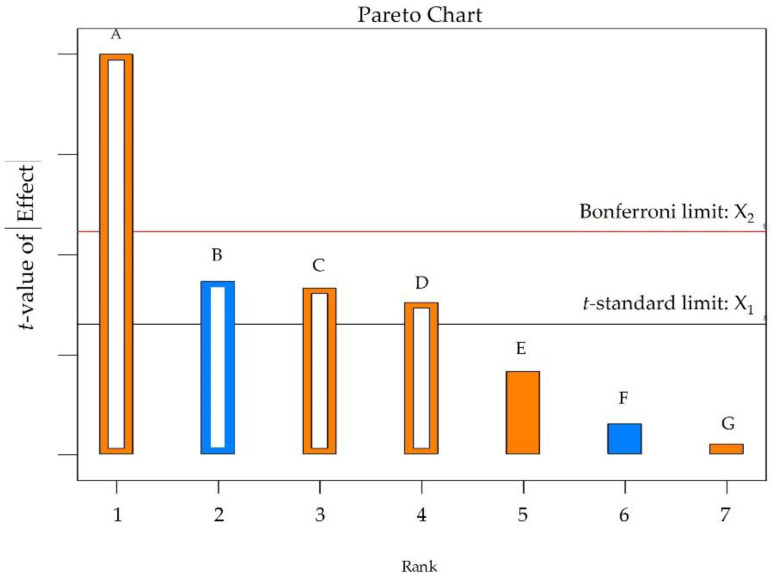
The Pareto chart for the principal effects of independent variables with the interaction of two variables. This example indicates that the model consists of seven variables (factors A–G). Among them, factors A, C, and D, represented by orange bars, demonstrate a significantly positive relationship with the response. On the other hand, factor B, depicted by a blue bar, exhibits a significantly negative relationship with the response. Factors E, F, and G, however, are considered not significant.

**Figure 3:**
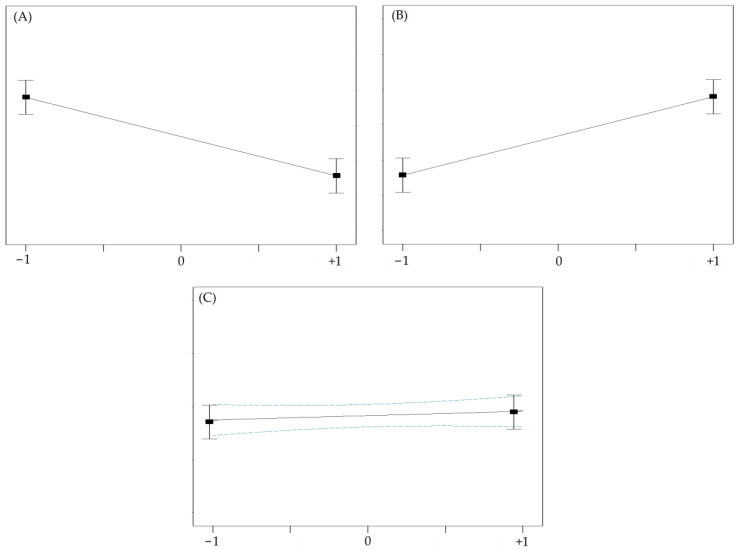
Illustrations of principal effect diagrams showing the relationship between the response variable and factor levels; the response variable increases as the level of the factor increases from a low (−1) to a high value (+1) (**A**). The response variable decreases as the factor’s value increases from a low level (−1) to a high level (+1) (**B**). The response variable holds constant as the level of the factor elevates from a low level (−1) to a high level (+1) (**C**).

As described previously, screening designs are commonly used in the initial step. The principal goal of the screening design is to identify and select significant parameters from a vast array of parameters. The Pareto charts are useful for identifying the most significant factors. The Bonferroni and *t*-standard *t*-value limits are included in the Pareto charts in order to assess the significance of the results [161]. On the basis of the bar graph (Figure 2), the fact that the *t*-limit of the factor is greater than the Bonferroni limit (highlighted in red line) indicates that the factor is unquestionably significant for the response. The effects above the *t*-standard limit (represented by the black line) with a Bonferroni limit below them indicate a possibly significant relationship, whereas the relationship is deemed insignificant when the effects are displayed below the *t*-standard limit. In addition, orange and blue bars typically represent positive and negative relationships. Figure 2 indicates, for instance, that factor A is the most significant parameter, followed by factor B, factor C, and factor D in that order. Nevertheless, factor E is an insignificant parameter. Parameters A, C, and D exhibit a positive correlation with the outcomes, whereas parameter B demonstrates a negative correlation with the responses. It is suggested that the main effect plot be utilized when analyzing the results. As shown in Figure 3, the response variable increases as the factor changes from a low (−1) level to a high level (+1) (Figure 3A), whereas the response variable decreases as the factor changes from a low (−1) level to a high level (+1) (Figure 3B). Therefore, the Pareto chart and main effect plots are useful for determining the significance of independent parameters on screening responses [162].

**Figure 4:**
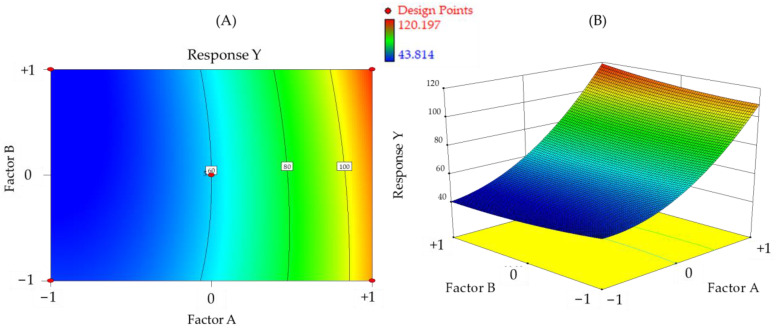
Contour (**A**) and 3D (**B**) response surface plots for analyses. The color variation helps in identifying regions of higher or lower values and discerning patterns or trends in the data. The color blue indicates a low value, while red is used to represent higher values. A color bar is provided to indicate the relationship between the response value and color.

After being screened by the screening designs, optimization is necessary. The data obtained from the analysis of experimental runs must be statistically calculated. The contour plot is a visual representation of the relationship between the response and the factor combinations. These data are presented as a two-dimensional (2D) plot (Figure 4A) depicting the relationship between the responses and/or numerical variables. Moreover, to visualize the relationship between a response variable and its independent variables, a three-dimensional (3D) surface plot (Figure 4B) is a type of graph that is useful for determining the optimal response values and operative parameters [163].

As stated previously, the outcomes of the experimental design should be statistically analyzed. ANOVA can determine whether or not the means of three or more groups differ. *F*-tests are utilized by ANOVA to statistically examine the equality of means. *F*-values and *p*-values are employed to determine the impact of factors on responses. *F*-values are utilized to support or reject the null hypothesis (H_0_). The null hypothesis is rejected if the estimated *F*-value (computed *F*-value) exceeds the crucial *F*-value. Nonetheless, the *F*-value alone is insufficient for the analysis; therefore, the *p*-value is required to validate the findings. If the *p*-value is less than 0.0500, the model terms are statistically significant, and the design is effective [164]. According to Table 6, factors A, B, C, and D have a statistically significant impact on the outcome.

The determination coefficient (R^2^) is the parameter used to measure the model’s fit quality. This value represents the proportion of response variation that can be predicted based on the independent variable. The range of R^2^ values is 0 to 1. When the R^2^ value reaches zero, the response variable cannot be explained or predicted by the independent variable. In contrast, if the R^2^ value is close to 1, the independent variable can predict the response variable [165]. When a model term is added, the R^2^ value is frequently increased. With a slight increase in R^2^, it is occasionally unnecessary to add new variables to the model. Therefore, it is suggested to calculate adjusted-R^2^ (R^2^-adj), a modified R^2^ value. When the significant term is added to the analyzed model, R^2^-adj increases. In contrast, R^2^-adj decreases as the non-significant term is incorporated into the model. The R^2^-adj value is typically less than the R^2^ value [166,167].

Lack of fit is used to explain whether a regression model lacks the ability to describe the relationship between the independent variable and the response variable. Therefore, for a model to be well-fitting, the lack of fit must not be statistically significant (*p* > 0.05). In addition, the adequacy precision (adeq. precision) of the model is utilized to calculate the signal-to-noise ratio. A model with a high adeq. precision value (greater than 4) is regarded as desirable [168].

**Figure 5:**
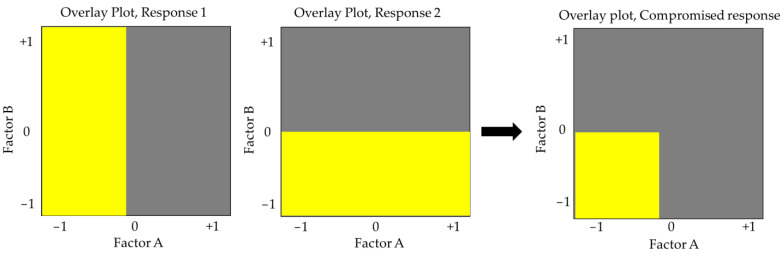
Design space is derived from the contour overlay plot, which consists of contour plots for each response laid on top of one another.

Following a thorough analysis of all parameters and ANOVA confirmation of a well-fitting model, the resulting model can be used to predict response when an independent parameter is within the experimental range. Multiple objectives make up the optimization process’s criteria. Maximum, minimum, targeted (desired) value and within-range determination are the possible objectives. Multiple responses require the identification of regions where requirements simultaneously provide the desired response property for the optimization process. By superimposing or overlaying contours of critical responses on a contour plot, it is possible to visually search for the optimal compromise. Through graphic optimization, the region of acceptable response values in the factor space is displayed. The regions that do not meet the optimization requirements are shaded in gray. Any region that is not gray satisfies the requirements for every response. Figure 5 illustrates that, unless the software’s default color settings have been modified, the region that meets the criteria will be highlighted in yellow, while the area that does not meet the criteria will be displayed in gray [169].

For a large number of response variables, the desirability function (D) is a statistically promising method. Its value varies between 0 and 1. The value of D reaching 1 represents the optimal condition for achieving the desired response values [169]. In conclusion, ANOVA is a useful technique that enables researchers to examine and investigate the numerous factors that influence response variables.

## 8. Application of Experimental Design

### 8.1. Experimental Design Applications in the UAE Procedure

Experimental design has been implemented in a variety of fields. Extraction is one of the topics that frequently employs experimental design in order to achieve the desired outcomes. Recent literature reviews are cited in Table 7 to illustrate the use of various experimental designs for the UAE method on various plant parts.

### 8.2. Experimental Design Applications in Electrospun Fiber Production

As stated previously, experimental design has been employed for a variety of purposes. Previous sections demonstrated that efficient extraction procedures can improve the properties of bioactive extracts. In addition, experimental methods can be utilized to enhance the quality of formulations derived from bioactive components, specifically electrospun fibers and nanofibers. Medicated nanofibers are fabricated from a solution or melt consisting of a carrier polymer and the desired bioactive extracts; these are typically processed by single fluid electrospinning [194]. Nanoscale fibers that mimic the native structure of the natural extracellular matrix could support the normal functions of cells, such as attachment and proliferation. Electrospinning generates nanofibers with exceptional characteristics, including a large specific surface area, high porosity, superior biocompatibility, and degradability. Consequently, electrospun nanofibers have been widely employed in the pharmaceutical industry as biosensors, medical implants, bioactive agent delivery systems, scaffolds for tissue engineering, and wound dressings [195]. They are useful for drug loading, amorphization of crystalline active ingredients, and storage stability extension [115]. In comparison to conventional wound dressings, the nanofibrous structures promote more effective wound healing. Nanofibers can optimally absorb exudates, provide a moist environment for cell respiration and proliferation, reduce bacterial infection, offer high permeability, and protect injured tissue from dehydration. Electrospinning can also incorporate pharmaceuticals and other bioactive molecules, such as growth factors, nanoparticles, antimicrobials, and anti-inflammatory agents, into nanofibers. Due to their high compatibility with blood and tissues, the electrospun biodegradable wound dressings promote healing and increase the rate of cell growth [196].

Numerous herbal extracts have been successfully encapsulated in electrospun fibers due to the fact that natural compounds exhibit biological activity in a nanoscale polymeric matrix. For instance, gelatin nanofibers loaded with *C. asiatica* extract accelerate wound healing by acting on the inflammatory and proliferative phases and preventing bacterial colonization of the injured site [197]. The effect of green tea extract in a matrix of chitosan and polyethylene oxide (PEO) electrospun fiber on *Escherichia coli* and *S. aureus* was observed with 4 mm and 6 mm inhibition zones, respectively [198]. Based on the antioxidative, antibacterial, and in vivo wound healing activities, in vitro release, and stability of *Garcinia mangostana* extract-loaded chitosan-ethylenediaminetetraacetic acid/polyvinyl alcohol (CS-EDTA/PVA) electrospun fibers, it was determined that the fibers could inhibit the growth of *S. aureus* and *E. coli* depending on the concentration of extract encapsulated within the fibers. In 11 days, skin re-epithelialization and hair follicle replacement of granulation tissue were observed in fibers loaded with 3% *w*/*w* extract. The fiber mats can retain up to 90% of α-mangostin (a biomarker in *G. mangostana* extract) for up to 3 months [199]. The electrospun gelatin nanofibers, containing 5% *w*/*v Curcuma comosa* Roxb. rhizome extract, exhibited significant DPPH antioxidant and anti-tyrosinase activity. In addition, antibacterial activity was observed against *S. aureus* and *Staphylococcus epidermidis*. Notably, the electrospun nanofibers of gelatin were able to stabilize the bioactive extract under accelerated storage conditions [200]. Natural extracts encapsulated in electrospun fibers to be applied in a variety of fields are achieving a very high rate of success, as evidenced by the preceding.

Today, there are numerous research findings on the production of electrospun fibers containing natural products. However, the number of articles reporting these fibers based on the optimization principle remains low [201]. Table 8 presents experimental designs from recent articles on electrospun fibers containing natural substances.

## 9. Discussion

Due to the concentration of this article on the use of DOE in the UAE process and electrospinning techniques for the extraction and encapsulation of natural bioactive extracts, the possibility of these topics will be discussed. UAE has several advantages over conventional extraction methods, including low energy consumption, less solvent usage, less extraction time, less active compound degradation, and high extraction yields. In addition, powerful UAE devices with a high production capacity are readily available on the market at reasonable prices. Due to these factors, UAE is currently prevalent in the botanical medicine and food industries. However, the UAE has a number of limitations that necessitate additional research before it can be utilized in a useful and effective manner. The majority of reports to date have centered on the optimization of UAE conditions for isolating plant-derived natural products. The optimal conditions in the UAE can be used to isolate bioactive compounds from raw natural materials on a large scale. In the herbal food and medicinal industries, UAE with optimization is anticipated to be widely used. UAE has been regarded as an environmentally favorable, uncomplicated, safe, cost-effective, and efficient extraction technique for obtaining bioactive components for the further development of electrospun fibers. The benefits of natural-product-containing electrospun fibers are well documented. However, DOE for optimizing the electrospinning procedure and enhancing formulation quality has not been extensively studied. Using DOE to enhance the efficacy of bioactive constituent extraction has become a promising method for optimizing the conditions required to achieve the goals of each investigation. In general, DOE can be divided into three steps: screening designs, which typically include 2-level full factorial design, fractional factorial design, and PBD; optimization designs, which typically include BBD and CCD; and verification processes, which are performed to validate the effectiveness of the obtained models. Taking this information into account, experimental design may be combined in various disciplines to achieve the desired outcomes, which include not only the highest extraction efficiency but also the highest product quality. This article’s findings are therefore applicable to the improvement and expansion of the extraction of natural products and the fabrication of electrospun fibers loaded with bioactive compounds, which are of immense importance to the herbal food and medication industries.

By sharing our research experience on using experimental designs to determine the optimal conditions for the preparation of natural product extracts from *K. parviflora* rhizomes, *S. alata* leaves, rusa deer velvet antlers, and beef adipose tissues, as well as for the fabrication of shellac fibers loaded with bioactive extracts by electrospinning technique, the authors hope that readers will be able to apply the new knowledge to a variety of fields of work.

## 10. Conclusions

Modern extraction techniques offer numerous advantages over traditional methods. However, by optimizing the extraction conditions using experimental design, it becomes possible to obtain extracts with the highest concentrations of active substances and biological activity. Ensuring quality control of the extracts, particularly through the use of validated methods to determine the contents of active substances, is crucial. Additionally, it is important to consider factors such as heavy metal contamination and the presence of pathogens. When preparing electrospun fibers containing bioactive extracts, numerous factors need to be considered, including the polymer solution, processing techniques, and environmental parameters. During natural product development, considering multiple parameters and conducting experimental trials are crucial. DOE plays a vital role in evaluating independent parameters, optimizing natural product extraction and formulation processes, and predicting extract and product performance. The DOE process involves three key steps: screening, optimization, and verification. When developing natural products, it is essential to consider various parameters, conduct experimental tests, and utilize DOE for evaluating independent parameters, generating design models, and predicting performance. Therefore, a three-step experimental design process is fundamental for achieving desired outcomes and improving natural product extraction and formulation processes.

## Figures and Tables

**Table 1:** The difference between ultrasonic bath mode and ultrasonic probe mode.

Topic	Bath Sonicator	Probe Sonicator
Characteristics of the tool	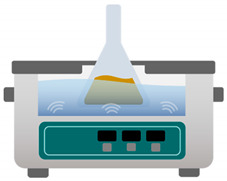	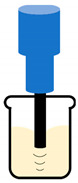
Direct/indirect contact method	A water bath is equipped to deliver energy to the samples. Thus, it is an indirect contact method.	A probe is inserted into the mixture of sample and solvent. Thus, it is a direct contact method.
The delivery of energy	A water bath equipped with transducers at the machine’s base transfers energy to the samples.	A probe is responsible for transferring energy to the samples.
Cross-contamination possibility	No	Yes
Number of samples per time	A large number of vessels simultaneously	One sample at a time

**Table 2:** Advantages and disadvantages of common conventional and non-conventional extraction methods.

Extraction Methods	Advantages	Disadvantage
**Conventional methods**		
Soxhlet Extraction [11,14,15]	A very simple technique Large quantities of plant materials can be extracted simultaneously No need to filter the solvent after extraction Can be used on both small and large scales	Probability of thermal decomposition of heat-labile substances Extensive extraction time Work intensive Permit constrained manipulation of variables Need a large amount of solvent
Maceration [15]	A simple method using non-complicated utensils and equipment Operator skill is not required Energy-saving process Because of the prolonged contact time, it is appropriate for substances that are very poorly soluble in the solvent A method suitable for less potent and cheaper extracts	In some instances, the extraction process can last for weeks Not entirely extracting the substance Very sluggish and time-consuming procedure More solvents are required
Percolation [11,15]	Less labor-intensive than maceration The extraction of thermolabile components is possible A method suitable for potent and expensive crude drugs Rapid and more thorough extraction	Longer duration than Soxhlet extraction More solvent uses A skilled individual is required Throughout the process, the particle size of the material should be given special consideration
Decoction [12,15]	Compatible with heat-resistant substances Expensive additional equipment is not required. Simple to execute No need for a trained operator	Not appropriate for the extraction of heat-sensitive components
**Non-conventional methods**		
Microwave-assisted extraction (MAE) [12,13,23,24]	Improve extract quality by enhancing bioactive compound purity and stability Enabling the use of fewer hazardous solvents Reduced administrative costs The very rapid rate of extraction Reduce power and solvent consumption	Specific equipment is required Low selectivity Depending on the solvent type and extraction temperature The reaction is inevitable at high temperatures
Supercritical fluid extraction (SFE) [12,23]	Increase the extraction rate. Greater selectivity of desired substances Low solvent consumption Automatic sample processing Superior extractive efficacy Due to the complete evaporation of CO_2_, a commonly used supercritical fluid, there are no solvent residues left in the extract	Quite expensive technology Require high pressures Unusual operating conditions Complicated phase behavior
Ultrasound-assisted extraction (UAE) [13,17,20,25]	Low power and solvent utilization Enhance extraction yield Reduced extraction time and lower temperatures	The heat generated during the extraction procedure can deteriorate heat-sensitive substances The effect of the ultrasound waves depends on the position of the container containing the matrix and solvent within the bath The inefficiency of energy transfer within the vessel containing the sample and solvent is a result of insufficient bath temperature and inadequate power control

**Table 3:** Main electrospinning parameters affecting nanofibers morphology.

Electrospinning Parameters	Effects of Different Parameters on the Diameter and Structural Morphology of Nanofibers
Flow rate	The polymer solution has sufficient time for polarization when the flow rate is modest, resulting in fibers with small diameters. If it is excessively high, rapid drying and minimum stretching result in the formation of bead fibers with large diameters [148].
Applied voltage	In general, a higher voltage favors the formation of fibers with smaller diameters, but it can also induce the ejection of more polymer fluid, resulting in fibers with larger diameters. When the voltage is increased beyond a critical value, the diameter initially decreases before increasing after a certain point. Increased repulsion forces account for the initial decrease in diameter [115].
Distance	The working distance between the spinneret and collector determines the stage of instability at which the jet is deposited on the collector. In order for the jet to fully extend and solidify, resulting in the formation of solid fibers, a sufficiently long distance is required. In fact, the long distance can cause fibers to become thinner. Jet solidification prevents the fiber from becoming thinner as the distance increases beyond a certain threshold. At a short distance, there is insufficient time for solvent evaporation. Consequently, nanofibers with flattened structures are produced. Beaded morphology occurs when the distance is insufficient [118].
Relative molecular mass	The molecular weight of a polymer has a significant effect on the rheological and electrical properties of electrospun solutions. Due to the limited chain entanglement, low molecular weight polymers tend to generate beads rather than fibers [117].
Viscosity	The low viscosity of the electrospun solution made it easier to produce thinner fibers. A high number of solvent molecules led to fewer chain entanglements and a lower density of surface charges, which allowed for the formation of beaded nanofibers. However, in the event that the viscosity was extraordinarily high, it would be difficult to expel the solution from the spinneret [118].
Surface tension	The reduced surface tension of the electrospun solution facilitates jet initiation. In general, decreasing surface tension enabled the production of thinner fibers and the gradual disappearance of beads [115].
Relative volatility	A polymer solution with a very high volatility is unsuitable for fiber spinning because the jet may solidify immediately upon exiting the spinneret. If the volatility is insufficient, the fibers will remain wet when they are deposited on the collector. The appearance of a porous microstructure is a result of increased volatility [117].
Conductivity	The high charge-carrying capacity of the electrospun solution results in a higher applied voltage due to its high conductivity. The higher the voltage, the smaller the diameter, and the wider the fiber diameter distribution [115].
Solubility of solvent	Critical to the formation of a homogeneous polymer solution is the solvent’s solubility, but a solvent with a high solubility does not necessarily produce an electrospinning-compatible solution. The volatility or vapor pressure of the solvent dictates its evaporation rate and, consequently, the jet’s rate of solidification [117].
Temperature	At a higher temperature, the polymer solution’s surface tension and viscosity decrease, allowing for the formation of thinner fibers. Nevertheless, at a higher temperature, the solvent evaporates more quickly, limiting the jet’s extension. The temperature has two contradictory effects on fiber diameter that must be carefully optimized [117].
Relative humidity	The relative humidity influences the solvent’s evaporation rate and the jet’s rate of solidification. Lower relative humidity encourages the formation of thinner, drier fibers. If the relative humidity is too low, the solvent evaporates rapidly, thereby limiting the extension of the jet. When the relative humidity reaches a certain level, however, water vapor in the air can enter the jet and cause morphological changes in the nanofibers [117].

**Table 4:** The number of treatments of fractional factorial design for 8 factors with 2-level [155,156].

*p*	Fraction	Number of Experiments
0 (full factorial design)	1	256
1	2	128
2	4	64
3	8	32
4	16	16

**Table 5:** Advantages and disadvantages of different screening designs [153].

Design	Advantages	Disadvantages
2-Level full factorial design	-Every possible combination of experimental runs is carried out. -The primary effects and their interactions are assessed.	-As the number of factors increases, the number of experimental runs increases exponentially.
Fractional factorial design	-A fractional factorial design requires fewer experimental iterations than a full factorial design.	-Certain interactions, particularly higher-order interactions that are insignificant compared to the main effects, can be disregarded by this design.
Plackett-Burman design	-With minimal runs, a large number of factors can be determined.	-This design is only useful for examining the main effects; interaction effects are not taken into account.

**Table 6:** An example of an ANOVA table.

Source	Sum of Squares	df	Mean Square	*F*-Value	*p*-Value	Conclusions	Remarks
Model	11037.31	9	1126.37	91.23	<0.0001	Significant	R^2^ = 0.9915
A	9524.77	1	9524.77	708.85	<0.0001	Significant	Adjusted-$R_{.}^{2}$ = 0.9807
B	1127.80	1	1127.80	83.89	<0.0001	Significant	Predicted-R^2^ = 0.8928 Adeq. precision = 28.34
C	168.39	1	168.39	12.53	0.0095	Significant	
D	70.66	1	70.66	5.26	0.0456	Significant	
E	29.05	1	29.05	2.16	0.1850	Not significant	
F	12.90	1	12.90	0.96	0.3600	Not significant	
G	8.07 × 10^−3^	1	8.07 × 10^−3^	6.01 × 10^−4^	0.9811	Not significant	
Residual	94.10	7	13.44				
Lack of fit	72.44	3	24.15	4.46	0.0914	Not significant	
Pure error	21.66	4	5.42				

**Table 7:** Applications of experimental design to the UAE method for various herbs.

Design *	Plant Materials	Ultrasound Source	Parameters	Levels	Optimal UAE Conditions	Responses/Results	Ref.
OFAT and CCD	*Moringa oleifera* L. leaves	Ultrasonic probe	-Frequency -Power -Solvent/solid -Temperature -Time -Extraction solvent	ND 80–240 W 25–40 mL/g 30–60 °C 5–25 min 52% *v*/*v* ethanol	ND 188 W 40 mL/g 52 °C 20 min 52% *v*/*v* ethanol	-The extract contained eight flavonoids with the highest concentrations of D-(+)-catechin, hyperoside, and kaempferol-3-*O*-rutinoside and had a higher total flavonoid content (TFC) and antioxidant activities (DPPH, ABTS, and FRAP) than those extracted by stirring-assisted extraction, Soxhlet-assisted extraction, and microwave-assisted extraction (MAE).	[170]
CCD	*Olea europaea* L. leaves	Ultrasonic bath	-Frequency -Power -Solvent/solid -Temperature -Time -Extraction solvent	28 kH_z_ 250 and 500 W 0.5–18.5 mL/g 15–75 °C 0–100 min 5–85 % *v*/*v* methanol	28 kH_z_ 250 W 12.8 mL/g 58.3 °C 71.25 min 61.75% *v*/*v* methanol	-The extract was superior to that obtained using the European Pharmacopoeia method in terms of oleuropein content, TPC (26.80 ± 1.96 mg GAE/g leaf), and antioxidant property (DPPH).	[171]
FFD and BBD	*Pistacia lentiscus* leaves	Ultrasonic bath	-Frequency -Power -Solvent/solid -Temperature -Time -Extraction solvent	39 kH_z_ 100 W 100–200 mL/g 30–50 °C 15–30 min 30–50% *v*/*v* ethanol	39 kH_z_ 100 W 130 mL/g 50 °C 15 min 50% *v*/*v* ethanol	-The extract contained the highest TFC (10.2 ± 0.8 mg/g).	[172]
BBD	*Pogostemon cablin* leaves	Ultrasonic bath	-Frequency -Power -Solvent/solid -Temperature -Time -Extraction solvent	30–50 kH_z_ ND 20–40 mL/g ND 10–20 min 100% *v*/*v* hexane	48.84 kH_z_ ND 26.99 mL/g ND 17.78 min 100% *v*/*v* hexane	-The optimal conditions resulted in the highest yield (182.24 mg/g) of a lipid-soluble extract with the highest patchoulol content (48.84%) and excellent fungal mycelial inhibition against virulent strains of *Aspergillus flavus* and *A. fumigatus* due to the disintegration of the matrix cell wall with numerous fractures, resulting in better phytochemical solubility in the extraction solvent (hexane). -To produce lipid-soluble extracts with a higher patchoulol content, the UAE has been favored over MAE and maceration with the same extraction solvent.	[173]
BBD	*Senna alata* leaves	Ultrasonic bath	-Frequency -Power -Solvent/solid -Temperature -Time -Extraction solvent	40 kH_z_ 160 W 20–40 mL/g 40–60 °C 10–20 min 95% *v*/*v* ethanol	40 kH_z_ 160 W 25.48 mL/g 59.52 °C 18.4 min 95% *v*/*v* ethanol	-The most rhein (10.44 mg/g DW) was found in the optimized extract, which was responsible for their antioxidant, anti-inflammatory, and antibacterial activities.	[107]
CCD	*Momordica charantia* fruits	Ultrasonic bath	-Frequency -Power -Sonication mode -Solvent/solid -Temperature -Time -Extraction solvent	ND ND normal and pulsed modes 0.1–0.5 mg/L 20–80 °C 10–15 min Deionized water	ND ND pulsed mode 0.25 mL/g 68.4 °C 12 min Deionized water	-The pulsed mode sonication yielded the best results in terms of optimal bioactive compound concentrations (antioxidant activity of 77.9%, total polyphenol content of 104.5 mg GAE/g, and total soluble protein content of 42.1 mg/1000 mL), which contributed to oxidative stress reduction.	[174]
BBD	*Aesculus hippocastanum* fruits	Ultrasonic bath	-Frequency -Power -Solvent/solid -Temperature -Time -Extraction solvent	40 kH_z_ 200 W 15–45 mL/g 30–60 °C 10–60 min 100% *v*/*v* methanol	40 kH_z_ 200 W 22.5 mL/g 60 °C 56.5 min 100% *v/v * methanol	-The UAE extraction yield was 21.683 ± 0.452% with a high concentration of pentadecanoic acid (19.34%), which was significantly higher than the Soxhlet extraction yield (19.455 ± 0.477%).	[175]
BBD	*Myrciaria cauliflora* fruits	Ultrasonic probe	Anthocyanins -Frequency -Power -Amplitude -Solvent/solid -Temperature -Time -Extraction solvent -Cycle -pH Phenolics -Frequency -Power -Amplitude -Solvent/solid -Temperature -Time -Extraction solvent -Cycle -pH	24 kH_z_ 200 W 30–70% 6.67–13.33 mL/g 10–70 °C 10 min 25–75% *v*/*v* methanol 2–7 s^−1^ 3–7 24 kH_z_ 200 W 30–70% 6.67–13.33 mL/g 10–70 °C 10 min 25–75% *v*/*v* methanol 2–7 s^−1^ 3–7	24 kH_z_ 200 W 34% 13.33 mL/g 39.8 °C 10 min 51% *v/v * methanol 0.47 s^−1^ 7 24 kH_z_ 200 W 68.5% 13.33 mL/g 26.0 °C 10 min 72% *v*/*v* methanol 0.50 s^−1^ 7	-The concentration of methanol was found to be the most influential variable in the extraction of anthocyanins and phenolics. Temperature and extraction cycle were additional variables that impacted anthocyanins. The optimal extraction time of 10 min was sufficient to extract both compounds quantitatively. -Due to their extreme temperature sensitivity, anthocyanins can be severely degraded during high-temperature extraction.	[176]
BBD	*Capsicum chinense* fruits	Ultrasonic bath	-Frequency -Power -Solvent/solid -Temperature -Time -Extraction solvent -pH	ND ND 25–75 mL/g 5–55 °C 5–15 min methanol: ethyl acetate (42: 58) 2–8	ND ND 72.5 mL/g 5.5 °C 5 min methanol: ethyl acetate (42: 58) 8	-UAE is sufficient, rapid, and efficient for extracting heat-sensitive capsinoids from peppers. -The capsiate content was found to be 1323 µg/g greater than in the previous report without optimization.	[177]
OFAT and CCD	*Moringa peregrina* seeds	Ultrasonic bath	-Frequency -Power -Solvent/solid -Temperature -Time -Extraction solvent	20 kH_z_ 348 W 5–20 mL/g 30–60 °C 5–30 min 80% *v*/*v* methanol	20 kH_z_ 348 W 17.8 mL/g 30 °C 26.3 min 80% *v/v * methanol	-The maximum yield of oil extraction was 53.101%, which was greater than the Soxhlet method yield of 43% after 11 h of extraction. -The UAE approach produced superior *M. peregrina* oil properties, including peroxide value, antioxidant activity (DPPH), total phenolic content (TPC), and iodine value (IV), compared to the Soxhlet method.	[178]
BBD	*Phaseolus vulgaris* seeds	Ultrasonic probe	-Frequency -Power -Amplitude -Solvent/solid -Temperature -Time -Extraction solvent -Cycle	26 kH_z_ 200 W 60–100% 20 mL/g 30 °C 10–30 min 40–80% *v*/*v* ethanol 2–7 s^−1^	26 kH_z_ 200 W 100% 20 mL/g 30 °C 10.3 min 46% *v/v * ethanol 4 s^−1^	-The extract contained the highest quantitative recovery of hydroxycinnamic acids, anthocyanins, and flavonols.	[179]
BBD	*Perilla frutescens* seeds	Ultrasonic bath	-Frequency -Power -Solvent/solid -Temperature -Time -Extraction solvent	ND 165–255 W 20–30 mL/g 30–50 °C 40–60 min 100% *v*/*v* water	ND 229 W 26 mL/g 43 °C 52 min 100% *v*/*v* water	-The yield of polysaccharides with substantial antioxidant activity was 6.14 ± 0.062%. -Scanning electron microscopy analysis revealed the formation of numerous holes on the surface of perilla seed meal after UAE.	[180]
CCD	*Abelmoschus esculentus* pulps	Ultrasonic bath	-Frequency -Power -Solvent/solid -Temperature -Time -Extraction solvent	40 kH_z_ 96–192 W 15–25 mL/g 40–70 °C 20–50 min 70% *v*/*v* ethanol	40 kH_z_ 142 W 25 mL/g 46 °C 40 min 70% *v*/*v* ethanol	-The UAE extract had a higher TPC (7.02 mg GAE/g dry weight) than the MAE extract (3.89 mg GAE/g dry weight) when extracted at the same temperature, time, solvent/solid ratio, and ethanol concentration. It also possessed exceptional abilities to scavenge free radicals and mitigate oxidative damage, which can be attributed primarily to hydroxycoumarin and quercetin derivatives.	[181]
BBD	*Citrus limetta* peels	Ultrasonic probe	-Frequency -Power -Amplitude -Solvent/solid -Temperature -Time -Extraction solvent -pH	40 kH_z_ 500 W 10–50% 30 mL/g 30–50 °C 10–30 min Water acidified with citric acid 1–3	40 kH_z_ 500 W 37% 30 mL/g 40 °C 24 min Water acidified with citric acid 1.9	-Pectin extracted under optimal conditions exhibited superior antioxidant, water/oil retention, emulsifying, and thermal properties compared to commercial pectin. UAE-obtained, extracted pectin may be utilized as a food ingredient.	[182]
BBD	*Citrus reticulata* Blanco cv. Sainampueng peels	Ultrasonic bath	-Frequency -Power -Amplitude -Solvent/solid -Temperature -Time -Extraction solvent	38.5 kH_z_ 30.34–59.36 W 10–50% 20 mL/g 30–50 °C 20–40 min 80% *v*/*v* acetone	38.5 kH_z_ 56.71 W 37% 20 mL/g 48 °C 40 min 80% *v*/*v* acetone	-Low power UAE has a higher extraction efficiency for total phenolic content (152.63 mg GAE/g DW) and hesperidin content (64.36 mg/g DW) than MAE at the same extraction temperature and time.	[183]
Factorial design	*Nymphaea lotus* stamens	Ultrasonic bath	-Frequency -Power -Solvent/solid -Temperature -Time -Extraction solvent	0–45 kH_z_ 400 W 10 mL/g 45 °C 20–60 min 50–100% *v*/*v* ethanol	34.65 kH_z_ 400 W 10 mL/g 45 °C 46 min 90% *v/v * ethanol	-The total flavonoid content was 235.45 mg/g dry weight, which was greater than the traditional heat reflux extraction yield of 169.64 mg/g dry weight.	[184]
BBD	*Phoenix dactylifera* L. Spikelets	Ultrasonic bath	-Frequency -Power -Solvent/solid -Temperature -Time -Extraction solvent	40 kH_z_ 110 W 100 mL/g 25–60 °C 20–40 min 25–50% *v*/*v* ethanol	40 kH_z_ 110 W 100 mL/g 40.80 °C 21.60 min 50% *v/v * ethanol	-Rutin and (+)-catechin were the major phenolic compounds in the extract under optimized UAE conditions. The total phenolic concentration in the optimized extract was 130.20 mg GAE/g DW, and DPPH radical inhibition was 87.20%.	[185]
BBD	*Derris reticulata* stems	Ultrasonic bath	-Frequency -Power -Solvent/solid -Temperature -Time -Extraction solvent	37 kH_z_ 120 W 10–30 mL/g 40–80 °C 20–60 min 100% *v*/*v* water	37 kH_z_ 120 W 10 mL/g 80 °C 60 min 100% *v*/*v* water	-The UAE extract contained gallic acid, *p*-coumaric acid, quercetin, and kaempferol in addition to high levels of phenolics (0.48 ± 0.03 mg GAE/g DW), flavonoids (0.15 ± 0.03 mg CE/g DW), and sugar (4.80 ± 0.65 mg/g DW) that can be used as a sweetener or sugar substitute in foods.	[186]
CCD	*Centella asiatica* L. aerial parts	Ultrasonic bath	-Frequency -Power -Solvent/solid -Temperature -Time -Extraction solvent	20 kH_z_ ND 10 mL/g 40–70 °C 30–90 min 40–80% *v*/*v* ethanol	20 kH_z_ ND 10 mL/g 48 °C 50 min 80% *v/v * ethanol	-The extract contained the greatest content of total triterpenoids, with 2.262 ± 0.046% *w*/*w* madecassoside, 1.325 ± 0.062% *w*/*w* asiaticoside, 0.082 ± 0.009% *w*/*w* madecassic acid, and 0.052 ± 0.007% *w*/*w* asiatic acid.	[187]
OFAT and BBD	*Andrographis paniculate* aerial parts	Ultrasonic probe	-Frequency -Power -Amplitude -Solvent/solid -Temperature -Time -Extraction solvent -Duty cycle	40 kH_z_ ND 10–100% 17 mL/g 70 °C 1–5 min 75% *v/v * ethanol 10–100%	40 kH_z_ ND 66% 17 mL/g 70 °C 5 min 75% *v/v * ethanol 11%	-The extract contained the highest concentration of andrographolide, 3.50 ± 0.17% *w*/*w*.	[188]
OFAT and BBD	*Anoectochilus roxburghii* whole plants	Ultrasonic bath	-Frequency -Power -Solvent/solid -Temperature -Time -Extraction solvent	ND 240–540 W 5–30 mL/g 10–60 °C 10–50 min 0–100% *v*/*v* methanol	ND 420 W 10.83 mL/g 35 °C 45 min 16.33% *v*/*v* methanol	-The extract had a high kinsenoside yield of 32.24% dry weight.	[189]
PBD and BBD	*Kaempferia parviflora* rhizomes	Ultrasonic bath	-Frequency -Power -Solvent/solid -Temperature -Time -Extraction solvent	40 kH_z_ 160 W 10–50 mL/g 30–80 °C 5–30 min 50–95% *v*/*v* ethanol	40 kH_z_ 160 W 50 mL/g 50 °C 15.99 min 95% *v/v * ethanol	-The highest concentration of total methoxyflavones was found in the extract at 327.25 mg/g.	[58]
BBD	*Allium cepa* L. bulbs	Ultrasonic probe	-Frequency -Power -Amplitude -Solvent/solid -Temperature -Time -Extraction solvent -pH -Cycle	20 kH_z_ 70 W 30–90% 50–100 mL/g 10–60 °C 10 min 50–100% *v*/*v* methanol 2–7 0.4–1 s^−1^	20 kH_z_ 70 W 85% 64 mL/g 58.8 °C 10 min 76.8% *v*/*v* methanol 2 0.94 s^−1^	-The optimization of UAE yielded the extract with the highest total flavonols content (8.78 ± 0.03 mg/g extract) and antioxidant activity (11.85 ± 0.11 mg trolox/g extract).	[190]
CCD and ANN	*Allium sativum* L. bulbs	Ultrasonic bath	-Frequency -Power -Solvent/solid -Temperature -Time -Extraction solvent	ND ND 10–30 mL/g 40–80 °C 10–30 min 20–100% *v*/*v* methanol	ND ND 20 mL/g 59 °C 13.5 min 71% *v/v * methanol	-The maximum values of the two output parameters found in the extract were 19.498 mg GAE/g fresh weight total phenolic content and 1.422 mg RUT/g fresh weight total flavonoid content.	[191]
CCD	*Thymus serpyllum* (a by-product from filter tea production)	Ultrasonic bath	-Frequency -Power -Solvent/solid -Temperature -Time -Extraction solvent	40 kH_z_ 42 W 20 mL/g 50–80 °C 40–70 min 45–75% *v*/*v* ethanol	40 kH_z_ 42 W 20 mL/g 70.28 °C 70 min 45% *v/v * ethanol	-In terms of yield (28.03%), TPC (4.39 mg GAE/g), and antioxidant activities (DPPH, ABTS, and FRAP), the extract was superior to the one obtained via the conventional process.	[192]
BBD	*Vaccinium vitis-idaea* L. fruit pomace	Ultrasonic bath	-Frequency -Power -Solvent/solid -Temperature -Time -Extraction solvent	50 kH_z_ 120–200 W 5–25 mL/g 40–60 °C 20–40 min 0–40% *v*/*v* ethanol	50 kH_z_ 166.86 W 20 mL/g 55.15 °C 35.54 min 40% *v/v * ethanol	-Anthocyanins are known to be sensitive to oxygen; therefore, antioxidants could be added to the extraction process to reduce oxidation. The UAE method with rosemary extract as an additive has been developed for extracting anthocyanins from *V. vitis-idaea* fruit pomace in order to increase extraction efficiency while reducing anthocyanin loss in the extract. The total concentration of anthocyanin was 4.12 ± 0.18 mg/g DW.	[193]

* OFAT–One-factor-at-a-time, PBD–Plackett-Burmam Design, FFD–Fractional Factorial Design, CCD–Central Composite Design, BBD–Box-Behnken Design, ANN–Artificial Neural Network; ND–No data.

**Table 8:** Applications of experimental design in the optimization of electrospun fibers containing natural substances.

Design *	Materials	Parameters	Levels	Optimization	Responses/Results	Ref
CCD	Polymer -AZG -PVA Bioactive substance -Catechin	-AZG concentration -PVA concentration -Voltage -Catechin -Feed rate -Needle-to-collector distance	2 g/L 80 g/L 16–20 kV 500–3000 mg/L 0.5–0.9 mL/h 10–14 cm	2 g/L 80 g/L 20 kV 3000 mg/L 0.5 mL/h 11 cm	-The average diameter of fibers containing and lacking catechin was 371 nm and 89 nm, respectively. -Catechin concentrations increased nanofiber loading capacity, changing their shape and microstructure. The polymer wall reacted with catechin hydroxyl groups as concentration increased. Hydrogen bonding and molecular chain adhesion increased nanofiber heat resistance. -Electrospun nanofibers loaded with catechin could be utilized in food packaging and pharmaceuticals.	[144]
CCD	Polymer -PVA Biocatalyst -β-glucosidase	-PVA content -Voltage -Feed rate -Needle-to-collector distance	8% *w*/*v* 12.5–13.5 kV 0.004–0.008 mL/min 12.5–17.5 cm	8% *w*/*v* 13 kV 0.006 mL/min 15 cm	-The average diameter of fibers was 343 nm. -β-Glucosidase was entrapped in PVA electrospun fibers using enzyme immobilization techniques to convert mogroside V in *Siraitia grosvenorii* fruit extract into siamenoside I. (sweetener).	[202]
BBD	Polymer -PLA Bioactive substance -Peanut protein isolate	-Solution mass fraction -Voltage -Feed rate -Needle-to-collector distance	10–14% *w*/*w* 14–18 kV 0.3–0.9 mL/h ND	10% *w*/*w* 16 kV 0.6 mL/h ND	-The average diameter of fibers was 164 nm. -Peanut protein isolate is comprised of eight essential amino acids. Because of their high porosity, large specific surface area, biocompatibility, and degradability, electrospun nanofiber membranes loaded with this isolate could be exploited for sustained drug release and wound dressing.	[203]
BBD	Polymer -Gelatin -PCL Bioactive substance -*Aloe vera* extract	-Gel content -Extract content -PCL content -Voltage -Feed rate -Needle-to-collector distance	5–15% *w*/*v* 0–10% *w*/*v* 12% *w*/*v* 15 kV 0.6–1.4 mL/h 15 cm	10% *w*/*v* 7.3% *w*/*v* 12% *w*/*v* 15 kV 1.2 mL/h 15 cm	-Fibers showed an average diameter of 125 nm with a tensile strength of 5.1 MPa. -The inclusion of *A. vera* extract enhanced antibacterial activity (>99.9% against Gram-positive bacteria and 85.63% against Gram-negative bacteria, respectively) and facilitated appropriate in vitro biodegradation. Moreover, the addition of *A. vera* extract enhanced cell viability without causing toxicity.	[204]
BBD	Polymer -PCL Bioactive substance -Propolis	-PCL content -Propolis content -Voltage -Feed rate -Needle-to-collector distance (cm)	8% *w*/*w* 0–7% *w*/*w* 12–18 kV 0.1–0.3 mL/h 10–17 cm	8% *w*/*w* 6.56% *w*/*w* 15.5 kV 0.238 mL/h 14.2 cm	-The average diameter of PCL/propolis fibers produced by electrospinning was 560 nm. -Electrospun fibers provided a slow release of antibacterial propolis, thereby preventing wound infection and accelerating the healing process.	[205]
BBD	Polymer -SHL Bioactive substance -*Kaempferia parviflora* rhizome extract	-SHL content -Extract content -Voltage -Feed rate -Needle-to-collector distance	36–40% *w*/*w* 1–5% *w*/*w* 12–24 kV 0.4–1.2 mL/h 20 cm	37.25% *w*/*w* 1.5% *w*/*w* 18 kV 0.8 mL/h 20 cm	-The fiber diameter was 574 nm with a low bead amount (0.48 beads/fiber). -Electrospun shellac fibers loaded with *K. parviflora* extract were able to produce a sustained-release profile within 10 h and demonstrated antibacterial activity against *S. aureus*. The optimized fibers can be developed into wound dressings.	[137]
BBD	Polymer -PCL -PVP Bioactive substance -*Lawsonia inermis* extract	-PCL content (% *w*/*v*) -PVP content (% *w*/*v*) -Voltage (kV) -Feed rate (mL/h) -Needle-to-collector distance (cm)	10–15% *w*/*v* 25–35% *w*/*v* 17 kV 1–2 mL/h 15 cm	12.5% *w*/*v* 30% *w*/*v* 17 kV 1.5 mL/h 15 cm	-The average fiber diameter was 241.17 nm with an air permeability of 1.63 × 10^3^ mL/s. cm^2^/mm. -The optimized dressing was effective against both *E. coli* and *S. aureus* without exhibiting any toxicity. Due to their excellent water vapor permeability, swelling ratio, and mechanical performance, PCL/PVP/*L. inermis* extract nanofibers are suitable as wound dressings for the prevention of infection and acceleration of wound healing.	[206]

* OFAT–One Factor at a Time, FFD–Fractional Factorial Design, CCD–Central Composite Design, BBD–Box-Behnken Design. AZG–Azivash gum, PVA–Polyvinyl alcohol, PLA –Polylactic acid, PCL–Poly ε-caprolactone, PVP–Polyvinylpyrrolidone, SHL–Shellac; ND–No data.

## Data Availability

The datasets used and/or analyzed during this study are available from the corresponding author upon reasonable request.
